# Metabolite Sequestration Enables Rapid Recovery from Fatty Acid Depletion in Escherichia coli

**DOI:** 10.1128/mBio.03112-19

**Published:** 2020-03-17

**Authors:** Christopher J. Hartline, Ahmad A. Mannan, Di Liu, Fuzhong Zhang, Diego A. Oyarzún

**Affiliations:** a Department of Energy, Environmental & Chemical Engineering, Washington University in St. Louis, St. Louis, Missouri, USA; b Warwick Integrative Synthetic Biology Centre & School of Engineering, University of Warwick, Coventry, United Kingdom; c School of Informatics, University of Edinburgh, Edinburgh, United Kingdom; d School of Biological Sciences, University of Edinburgh, Edinburgh, United Kingdom; Max Planck Institute, Marburg; Max Planck Institute for Terrestrial Microbiology

**Keywords:** metabolic dynamics, metabolic regulation, synthetic biology, transcription factor

## Abstract

Microbes adapt their metabolism to take advantage of nutrients in their environment. Such adaptations control specific metabolic pathways to match energetic demands with nutrient availability. Upon depletion of nutrients, rapid pathway recovery is key to release cellular resources required for survival under the new nutritional conditions. Yet, little is known about the regulatory strategies that microbes employ to accelerate pathway recovery in response to nutrient depletion. Using the fatty acid catabolic pathway in Escherichia coli, here, we show that fast recovery can be achieved by rapid release of a transcriptional regulator from a metabolite-sequestered complex. With a combination of mathematical modeling and experiments, we show that recovery dynamics depend critically on the rate of metabolite consumption and the exposure time to nutrients. We constructed strains with rewired transcriptional regulatory architectures that highlight the metabolic benefits of negative autoregulation over constitutive and positive autoregulation. Our results have wide-ranging implications for our understanding of metabolic adaptations, as well as for guiding the design of gene circuitry for synthetic biology and metabolic engineering.

## Abstract (precis)

Rapid metabolic recovery during nutrient shift is critical to microbial survival, cell fitness, and competition among microbiota, yet little is known about the regulatory mechanisms of rapid metabolic recovery. This work demonstrates a previously unknown mechanism where rapid release of a transcriptional regulator from a metabolite-sequestered complex enables fast recovery to nutrient depletion. The work identified key regulatory architectures and parameters that control the speed of recovery, with wide-ranging implications for the understanding of metabolic adaptations as well as synthetic biology and metabolic engineering.

## INTRODUCTION

Bacteria constantly adapt to changing environments by coordinating multiple levels of their intracellular machinery. Metabolic regulation provides a control layer that adapts metabolic activity to nutritional conditions. Such regulation relies on a complex interplay between gene expression and metabolic pathways (1). In the case of metabolic pathways, genes for nutrient uptake and consumption need to be upregulated when the specific nutrient is available in the environment. Failure to quickly increase pathway capacity may result in missed metabolic resource opportunity and a potential cost on fitness (2) and population survival (3–5). Conversely, upon nutrient depletion, the expression of specific metabolic enzymes can become wasteful and lead to a suboptimal use of biosynthetic resources (6, 7).

Metabolite-responsive transcription factors are a widespread regulatory mechanism in microbes. Upon sensing nutrient availability, they trigger changes in enzyme expression and metabolic flux (8). This strategy has been shown to control the dynamics of pathway upregulation in various ways (9–11). For example, negative autoregulation of transcription factors can speed the response time of gene expression (12), and feedback circuits based on metabolite-responsive transcription factors have been demonstrated to accelerate metabolite responses (13). While much of the literature has focused on the control of activation dynamics upon nutrient induction (14–16), little is known on how these regulatory mechanisms shape pathway recovery after depletion of nutrients.

**FIG 1:**
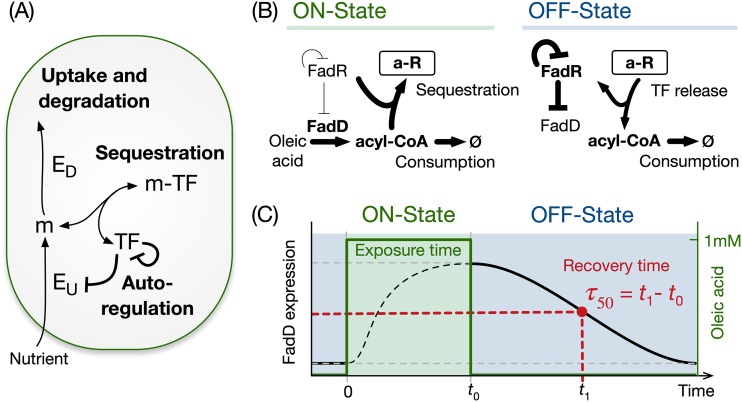
General architecture of a bacterial nutrient uptake system. (A) Regulation of nutrient uptake by a metabolite-responsive transcription factor, a ubiquitously observed control system in bacteria (Table S1). (B) We use the Escherichia coli fatty acid uptake as a model system. The on state is defined by induction at a constant level of oleic acid, which is imported as acyl-CoA by the uptake enzyme FadD. Acyl-CoA sequesters the transcription factor FadR, which derepresses expression of the uptake enzyme. The off state is defined by the washout of oleic acid after some time (*t*_0_) in the on state. The release of sequestered FadR recovers its repression on FadD synthesis. FadR is also subject to negative autoregulation. (C) Schematic of the experiments and simulations in this work, with defined exposure time to oleic acid (green area) and with recovery time of FadD levels in the off state (*τ*_50_) defined as the time to reach to halfway between the maximum and minimum concentrations.

Here, we study a common regulatory architecture found in over a dozen bacterial nutrient uptake systems (17) (Fig. 1A; see also Table S1 in the supplemental material). When a nutrient is absent from the environment, a metabolite-responsive transcription factor (MRTF) represses the expression of uptake and catabolic enzymes. When the nutrient is present, the nutrient is internalized and sequesters the transcription factor via reversible binding, thus preventing gene repression. This causes an upregulation of metabolic enzyme genes and an increase in the rate of nutrient import and utilization. A common feature of these control systems is the presence of negative autoregulation of the transcription factor (Table S1). After nutrient depletion, the MRTF must recover its repressive activity on the catabolic pathway genes to rapidly shut down pathway activity; yet, it is unclear which components of the regulatory system help accelerate the recovery dynamics.

10.1128/mBio.03112-19.6 TABLE S1 List of metabolite-responsive transcription factors (TFs) that control the expression of nutrient uptake enzymes in Escherichia coli, taken from the EcoCyc database. All of these systems follow the schematic as our model system illustrated in Fig. 1A. Download Table S1, DOCX file, 0.1 MB. Copyright © 2020 Hartline et al. 2020 Hartline et al. This content is distributed under the terms of the Creative Commons Attribution 4.0 International license.

Using the Escherichia coli fatty acid catabolic pathway as our model system, we took a theoretical-experimental approach to study its recovery dynamics in response to a nutrient shift from an on state to an off state. As illustrated in Fig. 1B, these two states are defined as an environment with and without the presence of oleic acid as a carbon source, respectively. In the on state, oleic acid is imported as fatty acyl coenzyme A (acyl-CoA), which binds to the transcription factor FadR and sequesters it into a complex. This acyl-CoA sequestration releases FadR from its cognate DNA elements (18), which relieves the repression of the uptake gene *fadD* and thus accelerates the import of oleic acid. We found that upon the depletion of oleic acid, repression by FadR is recovered via its rapid release from the sequestered complex, which in turn is driven by the consumption of acyl-CoA. We further found that the architecture of FadR autoregulation affects the maintenance of a sequestered pool of FadR. In particular, negative autoregulation enables a large sequestered transcription factor (TF) pool during the on state and, at the same time, a reduced biosynthetic cost in the off state. Our results shed light on the regulatory mechanisms that allow cells to rapidly adapt to environmental shifts and provide insights for the design of gene circuits in synthetic biology and metabolic engineering applications, particularly where strain performance is sensitive to nutrient fluctuations and inhomogeneities typical of large-scale fermentations.

## RESULTS

### Recovery dynamics in the fatty acid uptake system.

To study the recovery dynamics of fatty acid uptake, we built a kinetic model based on four core components of the regulatory system, FadD (*D*), free FadR (*R*), acyl-CoA (*A*), and sequestered FadR (aR). The model represents cells growing at a fixed growth rate with oleic acid at a fixed concentration in the medium. We simulated the recovery dynamics by mimicking the following three stages in our experimental setup: preculture without oleic acid, response to induction in the on state, and recovery in the off state. During preculture, we ran the model to steady state in the absence of oleic acid and then initiated simulations of the on state from the steady state achieved in preculture, with a fixed concentration of oleic acid for a defined exposure time. The concentrations achieved at the end of the on state were used as initial conditions for the off state, which was simulated without oleic acid until the system recovers to the steady state in preculture (Fig. 1C).

We defined two metrics to quantify the recovery dynamics after the switch from the on to the off state (Fig. 1C). First, we define the recovery time as the time taken for FadD to decrease to halfway between its maximum and minimum steady-state value after nutrient depletion (*τ*_50_) (Fig. 1C). Second, we defined the metric *η* as the proportion of free FadR released from the sequestered complex after one doubling time, shown in equation 1:

(1) $$\eta =\frac{{\text{FadR}}_{\text{DT}}-{\text{FadR}}_{\text{DT-new}}}{{\text{FadR}}_{\text{DT}}}$$

where FadR_DT_ and FadR_DT-new_ are the concentrations of free FadR and newly expressed FadR in the off state after one doubling time (DT). This definition allows us to quantify the contribution of free FadR released from the sequestered pool to the recovery dynamics.

**FIG 2:**
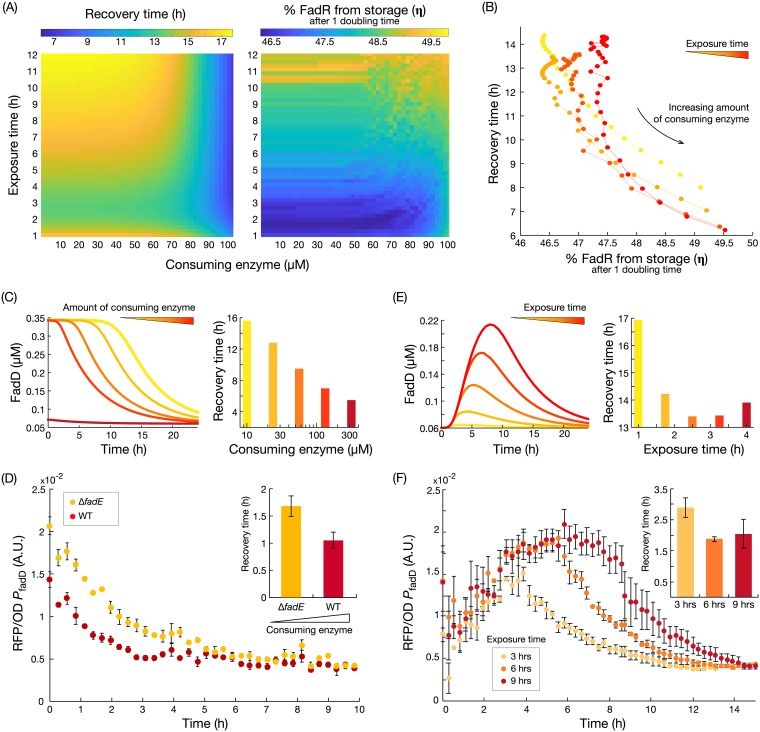
Nutrient exposure time and speed of metabolite consumption in the off state shape the recovery time. (A) Predicted recovery time (*τ*_50_) and proportion of free FadR released from sequestration after one doubling time (*η*) for variations in the amount of consuming enzyme and nutrient exposure time. (B) Inverse relation between the proportion of released FadR (*η*) and the predicted recovery time. (C) Simulated time course of FadD concentration during the off state and predicted recovery times for increasing concentrations of acyl-CoA consuming enzyme. (D) Measured time course of *fadD* expression when switching from an on to off state for strains with low (Δ*fadE* mutant reporter) and high (WT reporter) concentrations of acyl-CoA-consuming enzyme. Strains were switched from M9G plus 1 mM oleic acid to M9G medium at time zero. Error bars represent standard error of the mean (SEM) of the results from biological triplicates (*n* = 3). Recovery times were calculated from exponential fits to each of the triplicate time course data (inset). Error bars represent the SEM of the results from biological triplicates (*n* = 3). (E) Time course simulations of FadD induction and recovery dynamics, and predicted recovery times, for increasing exposure times. (F) Measured time course of *fadD* expression from the WT reporter strain grown for 3, 6, and 9 h of exposure to oleic acid (M9G plus 1 mM oleic acid) and then switched to an off state (M9G). Error bars represent the SEM of the results from biological triplicates (*n* = 3). Recovery times were again calculated from exponential fits, with error bars indicating the SEM of the results from triplicates (*n* = 3).

Since pathway recovery depends on the system state at the time of the on to off switch, we used the kinetic model to study the relation between the initial conditions at the time of the switch and the recovery dynamics. To this end, we studied the impact of exposure time to oleic acid during the on state, as well as the amount of acyl-CoA-consuming enzyme. We simulated the off-state dynamics for 2,500 combinations of 50 acyl-CoA-consuming enzyme concentrations and 50 exposure times and calculated the *τ*_50_ and *η* for each. The simulation results of the off-state dynamics (Fig. 2A) suggest that the *τ*_50_ decreases with increasing concentrations of consuming enzyme, while the amount of released FadR (*η*) increases with both the consuming enzyme and the exposure time. Further simulations suggest that when exposure time increases, the pool of acyl-CoA accumulates further, with a rise time from 8.5 to 10 h, for levels of consuming enzyme between 100 μM and 6 μM (Fig. S2B). This larger pool takes a longer time to be consumed in the off state (Fig. S2A) and so delays the release of FadR from the complex. This results in a longer recovery time (details in Text S1 and Fig. S2). Model simulations also reveal a strong inverse relation between *τ*_50_ and *η* (Fig. 2B), indicating that the release of FadR from sequestration by acyl-CoA provides a mechanism for cells to achieve rapid recovery during nutrient depletion. Further, the sensitivity of this inverse relation increases when cells are exposed to a longer on state. Simulations show that longer cell exposure times to oleic acid increase the pool of sequestered FadR (Fig. S2). Consequently, in the off state, more FadR can be released from sequestration than with new FadR synthesis, thus increasing the sensitivity of *τ*_50_ changes in the amount of released FadR.

10.1128/mBio.03112-19.1 TEXT S1 Details on experimental methods, model fitting, and mathematical analysis. Download Text S1, DOCX file, 0.8 MB. Copyright © 2020 Hartline et al. 2020 Hartline et al. This content is distributed under the terms of the Creative Commons Attribution 4.0 International license.

To verify the model predictions, we sought to experimentally perturb *η* through two complementary strategies, as follows: (i) by engineering strains with different amounts of acyl-CoA consuming enzymes and (ii) by manipulating the exposure time to oleic acid. We first constructed a reporter strain with a decreased consumption rate of acyl-CoA, the Δ*fadE* mutant reporter strain (see Table S3B), where we deleted the *fadE* gene encoding the second step of the fatty acid β-oxidation pathway. This prevents metabolization of acyl-CoA by β-oxidation and leaves membrane incorporation (catalyzed by enzyme PlsB) as the only pathway for acyl-CoA consumption. We measured *fadD* expression dynamics after switching the strains from the on state (M9G plus 1 mM oleic acid medium) to the off state (M9G medium) using a red fluorescent protein (RFP) reporter fused downstream of the *fadD* promoter. The *fadE* knockout strain displayed a slower recovery than did the wild type, with a ∼60% increase in recovery time (Fig. 2D), confirming our theoretical prediction shown in Fig. 2C. The measured increase in recovery time entails an increased expenditure of biosynthetic resources to import a metabolite that is no longer present in the environment.

Next, we measured the *fadD* recovery dynamics after switching the cultures from growth with 3, 6, and 9 h of exposure time in the on state. As predicted from the model in Fig. 2E, the measured recovery time decreased for an increase in exposure time (Fig. 2F). However, we observe that recovery time is not decreased further beyond 6 h of exposure to oleic acid. We speculate that faster recovery is counteracted by the delay of having to consume a higher level of accumulated acyl-CoA or because the maximum level of sequestered FadR may already have been achieved at 6 h.

### Impact of autoregulatory architecture on recovery dynamics.

**FIG 3:**
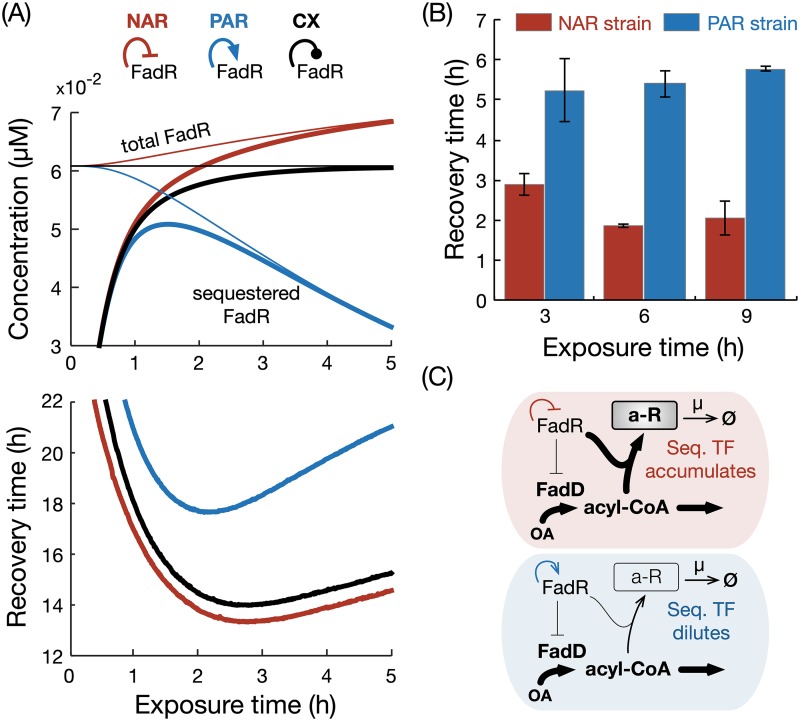
Impact of regulatory architecture on the recovery time after nutrient depletion. (A) Top, simulated steady-state concentrations of sequestered (thick lines) and total (thin lines) FadR for various times spent in the on state for three regulatory architectures of FadR; constitutive expression (black line) is represented by a blunt line. Bottom, predicted recovery times for each architecture. (B) Measured recovery times in the WT (WT reporter) and positively autoregulated strain (PA reporter) (Tables S3B and S4) for 3, 6, and 9 h of exposure in on state. Recovery times were calculated from exponential fits to each of the triplicate time course data (see File S1 and Fig. S3), and error bars represent the SEM of the calculated values (*n* = 3). (C) Schematics illustrating how negative and positive autoregulation affect the buildup of sequestered (Seq.) FadR in the on state.

Among the uptake systems in E. coli with the architecture of Fig. 1A, we found that the majority have a transcriptional regulator that represses its own expression, few systems have constitutive expression of the regulator, and no systems display positive autoregulation (see Table S1). To better understand the salient features of each regulatory architecture and how they affect recovery dynamics, we built variants of our kinetic model with FadR under constitutive expression and positive or negative autoregulation (details in Materials and Methods). Simulations of the recovery dynamics in the off state for various exposure times in the on state suggest that these architectures behave similarly for short exposure times (<1 h), quickly sequestering all of the free FadR (Fig. 3A, top). For longer exposure times (>1 h), model simulations suggest important differences in the dynamics of sequestered FadR among the various modes of autoregulation. Negative autoregulation shows an accumulation of sequestered FadR, while positive autoregulation leads to an overall depletion of sequestered FadR. Constitutive expression causes the total level of sequestered FadR to be maintained at a constant level (Fig. 3A).

To elucidate whether these predicted trends are a consequence of the model parameters or are inherently determined by the autoregulatory architecture, we analyzed the model and found relationships for the change in steady-state concentrations of total FadR (Δ*R_T_*) in each autoregulatory architecture (the details of the derivation are in Text S1), as follows: negative autoregulation, Δ*R_T_* > 0; positive autoregulation, Δ*R_T_* < 0; constitutive expression, Δ*R_T_* = 0. These relationships are valid for any combination of positive parameters, and therefore, the long-term trends observed in Fig. 3A are structural properties of the model.

To determine the effect of the three regulatory architectures on the recovery time, we simulated the recovery dynamics of each architecture for various exposure times and calculated the recovery time (Fig. 3A, bottom). We observe that the overall relationships between recovery time and exposure time are similar across the three architectures (Fig. 3A, bottom inset). However, for positive autoregulation, we found recovery to be significantly slower for a wide range of exposure times. To test this prediction, we engineered an E. coli strain with positively autoregulated FadR expression by replacing the native *fadR* promoter with one that activated by FadR (P*_fadRpo_*) (see Table S4) and a P_fadD_ reporter plasmid. The positively autoregulated reporter strain (PA reporter) (Tables S3B and S4) was grown in the on state (M9G medium plus 1 mM oleic acid) and then rapidly switched to the off state (M9G medium) after 3, 6, and 9 h. We measured the *fadD* expression dynamics (see time course dynamics in Fig. S3B) and calculated the respective recovery times (Fig. 3B). Consistent with the trend predicted from the model, the recovery times for the positively autoregulated strain increased with the exposure time of oleic acid in the on state (Fig. 3C).

### Negative autoregulation provides a resource-saving recovery strategy.

**FIG 4:**
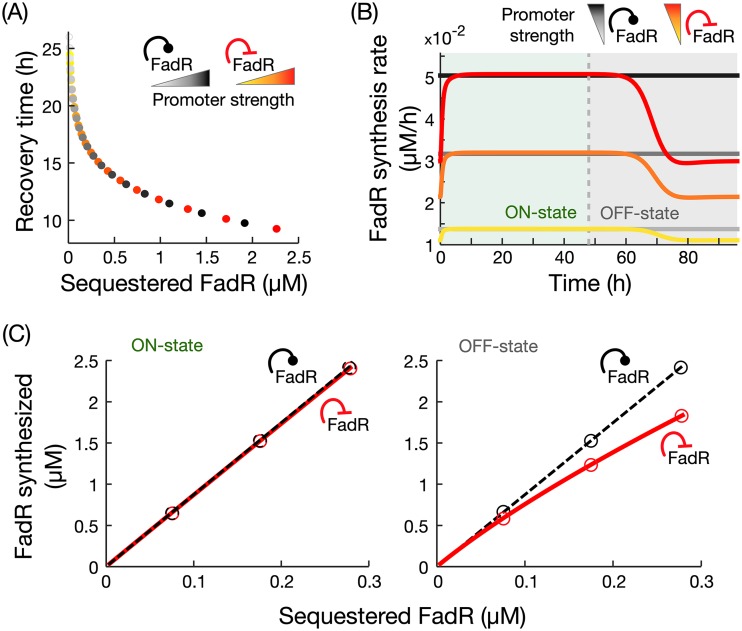
Comparison of recovery dynamics in constitutive expression and negative autoregulation. (A) Simulated recovery times for variations in the strength of FadR’s own promoter, with the two architectures achieving the same recovery times. (B) Time course simulations of FadR synthesis rates for 48 h in the on state (1 mM oleic acid) and off state, for increasing promoter strengths; the yellow curve represents the response with the fitted promoter strength value (Table S2B). To ensure fair comparison, promoter strengths were chosen to achieve the same recovery times in the two architectures. (C) Cost of FadR synthesis for increasing concentrations of sequestered FadR, modified by changes to *fadR* promoter strength. Circles correspond to costs associated with simulations shown in panel B. Details of the simulations are in Materials and Methods.

The results from the above-described autoregulation relationships suggest that constitutive expression and negative autoregulation can both maintain large amounts of sequestered FadR for long exposure times to oleic acid. Our earlier results showed that longer exposure times lead to a larger pool of sequestered FadR (Fig. S2D), which enables a faster recovery time (Fig. 2E and F). We thus asked which system parameters influence the steady-state pool size of sequestered FadR in these two architectures. We found that for high concentrations of oleic acid, the steady-state concentration of sequestered FadR in the on state is given by equations 2 and 3 (details in Text S1):

(2) $$\text{negative autoregulation}:\underset{A\to \infty }{\text{lim}}\text{ aR}=\frac{a_{n}}{\text{μ}},$$

(3) $$\text{constitutive expression}:\underset{A\to \infty }{\text{lim}}\text{ aR}=\frac{p_{c}}{\text{μ}},$$

where *A* and aR are the steady-state concentrations of acyl-CoA and sequestered FadR, respectively, and *a_n_* and *p_c_* are the promoter strengths in each case. These results suggest that at high oleic acid concentrations, the amount of sequestered FadR scales linearly with the strength of its own promoter. In simulations of both architectures in the on state induced with high concentration of oleic acid (1 mM) and various promoter strengths, we found that increasing promoter strength both increases the amount of sequestered FadR in the on state and decreases the recovery time (Fig. 4A).

10.1128/mBio.03112-19.7 TABLE S2 (A) Parameters of the kinetic model. (B) Results of parameter fitting. Shown are optimal parameter values together with search bounds and summary statistics for 100 independent fits. The bounds on growth rate, μ, are based on ±2 the SEM from data (shown in Fig. S2A, inset). Hill coefficients are fixed to *n_R_* of 1 and *n_D_* of 2, based on the number of FadR binding sites on the *fadR* and *fadD* promoters. The concentration of PlsB is fixed to 0.1369 μM, as taken from Schmidt et al. (2016), Nat. Biotechnol, 34(1). Download Table S2, DOCX file, 0.1 MB. Copyright © 2020 Hartline et al. 2020 Hartline et al. This content is distributed under the terms of the Creative Commons Attribution 4.0 International license.

10.1128/mBio.03112-19.8 TABLE S3 (A) Plasmids used in this study. (B) Strains used in this study. Download Table S3, DOCX file, 0.1 MB. Copyright © 2020 Hartline et al. 2020 Hartline et al. This content is distributed under the terms of the Creative Commons Attribution 4.0 International license.

10.1128/mBio.03112-19.9 TABLE S4 Sequences of engineered promoter with positively autoregulated *fadR*. (A) Native *fadR* promoter sequence (P*_fadR_*). Bold lettering indicates an FadR operator site, and blue lettering indicates a coding sequence. (B) Positively autoregulated *fadR* promoter (P*_fadR_*_po_) engineered in this work. The underlined sequence is derived from the *fabA* promoter region of E. coli DH1 genome. (C) Engineered P*_fadR_*_po_ used to control *rfp* expression. Download Table S4, DOCX file, 0.1 MB. Copyright © 2020 Hartline et al. 2020 Hartline et al. This content is distributed under the terms of the Creative Commons Attribution 4.0 International license.

The results shown in Fig. 4A also suggest that through tuning of *fadR* promoter strength, in principle, constitutive expression and negative autoregulation can produce the same recovery time. We thus sought to identify the potential benefits of one architecture over the other in terms of the recovery dynamics in the off state. Since the production of FadR entails a biosynthetic cost, we compared both regulatory architectures in terms of the cost of FadR synthesis. From time course simulations of FadR synthesis rates in the on and off states (Fig. 4B), we computed the total amount of synthesized FadR for increasing *fadR* promoter strengths by integrating the area under the curves (Fig. 4C). Our results show that both architectures require identical biosynthetic costs for FadR in the on state, but negative autoregulation leads to a reduced biosynthetic cost for FadR in the off state compared to constitutive expression (Fig. 4C).

## DISCUSSION

In this paper, we combined mathematical modeling and experiments to study metabolic pathway recovery upon depletion of an external nutrient. Changes in nutrient conditions trigger transcriptional programs that adapt cell physiology (19) to meet the cellular energy budget (20). We chose the regulation of fatty acid uptake in E. coli as our model system, as it is representative of a widely conserved transcriptional program for controlling the uptake of nutrients in bacteria (see Table S1 in the supplemental data). We show that fast recovery after nutrient depletion can be achieved by a rapid release of a transcriptional regulator from a metabolite-sequestered complex. In particular, a sizable contribution of FadR rapidly made available after oleic acid depletion came from its release from its sequestered complex form (aR), as opposed to new synthesis. The rapid availability of FadR quickly recovers its inhibition on the *fad* regulon and so shortens the recovery time. Furthermore, our model simulations and experiments have demonstrated that increasing the amount of FadR stored in complex form during nutrient exposure and fast consumption of acyl-CoA (the sequestering metabolite) facilitate a speedy recovery in the off state.

Our model simulations show that pathway recovery is delayed by high intracellular acyl-CoA concentrations, which slow the release of free FadR from stored complex until those high concentrations are reduced. This delay occurs because FadR is only able to sense the intracellular metabolite concentrations, which can remain high even when extracellular metabolite concentrations are low. During this delay, wasteful expression of the uptake pathway continues despite the absence of oleic acid in the environment. Previous research has shown that upon nutrient induction, metabolite dynamics tend to lag behind slow upregulation of metabolic enzymes (13). In contrast, here we find that after inducer depletion, the recovery of metabolic enzymes back to their downregulated state lags behind the metabolite dynamics. This has important implications for designing synthetic control circuits which utilize nonmetabolizable inducers such as isopropyl-β-d-thiogalactopyranoside (IPTG) or methyl-β-d-thiogalactopyranoside (TMG). Without consumption of the inducer, the postinduction recovery response will be slow and may cause a dramatic drain of cellular resources. Our simulations of the relation between sequestered FadR and recovery time suggest that this inherent lag can be compensated for by storing and releasing larger amounts of TFs, which highlights the benefits of maintaining a sequestered pool of FadR.

Further mathematical analyses revealed principles that explain how autoregulation shapes the recovery time. We found that systems with only negative autoregulation and constitutive expression can maintain the pool of sequestered FadR needed for a rapid recovery. In contrast, we found that positive autoregulation loses this storage over time, resulting in a reduced availability of FadR after nutrient depletion and slower recovery times. We additionally found that negative autoregulation of the transcription factor reduces the total biosynthetic cost of for FadR in a full on-off-state cycle compared to using constitutive expression. This occurs because both systems need to maintain the same level of sequestered FadR in the on state in order to achieve the same recovery time, but only negative autoregulation allows FadR synthesis to be downregulated in the off state. Thus, negative autoregulation provides a resource-saving strategy for controlling the recovery dynamics compared to constitutive expression. We found that the transcriptional regulators in 13 out of 18 nutrient uptake systems (see Table S1) have negative autoregulation, suggesting an evolutionary pressure for a resource-saving control strategy. Past studies in the literature have found that expression under negative autoregulation can decrease response times in gene expression (12), linearize the dose response in responsive systems (21), and even speed up metabolic dynamics (13). In addition to these properties, we find that negative autoregulation enables a rapid and more resource-saving metabolic recovery to nutrient depletion.

Recent efforts in synthetic biology focus on engineering gene control circuits to manipulate microbial metabolism (22–24). One key goal of such control systems is to rapidly turn off metabolic pathways in response to metabolic signals (25–27). Our results provide core design principles for engineered metabolic systems with a tunable response to nutrient depletion, which could be used as a pathway control tool in bioreactors. Our experiments and simulations reveal that the recovery time can be simply tuned through well-established promoter engineering techniques (28–30). Further, we identified regulatory architectures with differing dynamic responses to nutrient depletion, which provides further avenues for the tuning system response to the highly dynamic and heterogeneous environments typical of large-scale fermenters. These design rules can be readily applied to mitigate against deleterious nutrient fluctuations found in metabolic engineering applications.

## MATERIALS AND METHODS

### Materials.

Phusion DNA polymerase, T4 DNA ligase, restriction enzymes, and Teknova 5× M9 minimal salts were purchased from Thermo Fisher Scientific (Waltham, MA, USA). Gel purification and plasmid miniprep kits were purchased from iNtRON Biotechnology (Lynnwood, WA, USA). Oligonucleotides were synthesized by Integrated DNA Technologies (Coralville, IA, USA). All other reagents were purchased from Sigma-Aldrich (St. Louis, MO, USA).

### Plasmids, strains, and genome modifications.

A list of plasmids used along with promoter sequences in this study is provided in Text S1 and Tables S3 and S4 in the supplemental data. E. coli DH10β was used for plasmid construction. The plasmid pSfadDk-rfp was constructed by cloning the *fadD* promoter (500 bp upstream of its translation start site) into the 5′ end of the *rfp* gene in a BglBrick vector, pBbSk-rfp (31), using Golden Gate DNA assembly (32). The positively autoregulated *fadR* strain was engineered by replacing *fadR*’s native promoter with the FadR-activated promoter P*_fadRpo_* via CRISPR-Cas9 genome editing (33). Detailed engineering methods and the characterization of the P*_fadRpo_* promoter are described in Text S1.

Three reporter strains were created to measure expression dynamics from the *fadD* promoter. These strains were created by transforming plasmid pSfadDk-rfp into either the wild-type DH1 strain, DH1 Δ*fadE*, or an engineered strain with positively autoregulated *fadR*, resulting in the wild-type (WT) reporter, Δ*fadE* mutant reporter, and PA reporter, respectively.

### Medium conditions.

All strains were grown from single colonies and cultivated overnight in Luria-Bertani (LB) medium before the experiments. For off-state culture conditions, cells were grown in M9 minimal medium (34) supplemented with 1% glycerol and 0.5% Tergitol NP-40 solution (M9G). For on-state culture conditions, cells were grown in M9G plus 1 mM oleic acid (M9G+OA). All cultures were supplemented with appropriate antibiotic selection (50 mg/liter kanamycin, 100 mg/liter ampicillin).

### Assays of *fadD* expression dynamics.

To measure the recovery dynamics, reporter strains were grown in 3 ml M9G+OA for 24 to 48 h at exponential-growth state. To rapidly switch nutrients, cells were centrifuged (5,500 relative centrifugal force [rcf], 2 min) and washed twice in M9G. Cultures were then diluted in M9G medium to an optical density at 600 nm (OD_600_) of 0.08 and transferred to a Falcon 96-well imaging microplate (Corning, NY, USA). The microplate was then incubated in an Infinite F200 Pro plate reader (Tecan, Männedorf, Switzerland) at 37°C with constant shaking. To maintain exponential growth during measurement, cultures were diluted by a factor of 5 for three times during incubation. Kinetic measurements of cell density (absorbance at 600 nm) and RFP fluorescence (excitation, 584 ± 9 nm; emission, 620 ± 20 nm) were taken every 900 s until all diluted cultures reached stationary phase. Fluorescence from water in the same 96-well plate was used as the background and was subtracted from all fluorescence measurements. The background-corrected fluorescence was later normalized by cell density. To calculate the recovery time, the average of three biological replicates were fitted to an exponential curve, shown in equation 4:

(4) $$F=a\cdot e^{-b\cdot t}+c$$

where *F* is the background-corrected, cell-density-normalized fluorescence. The recovery time was calculated as *τ*_50_ = log_2_/*b*.

For switches after defined times in the on state, cultures were first grown in exponential-growth phase for 24 to 28 h in M9G. Samples from these cultures were then centrifuged (5,500 rcf, 2 min) and suspended in M9G+OA with an initial OD_600_ of 0.08 and cultivated in 96-well plates for various amounts of time as indicated.

### Kinetic model of fatty acid uptake.

To study the dynamic response to oleic acid exposure (on state) and its recovery (off state) (Fig. 1C), we built a kinetic model of the fatty acid uptake system. We define the model as a system of ordinary differential equations (ODEs) describing the rate of change of each species, shown in equations 5 to 8:

(5) $$\frac{\mathrm{dR}}{\mathrm{dt}}=P_{R}(R,p_{r})-k_{f}\cdot R\cdot A^{2}+k_{r}\cdot \text{aR}-\text{μ}\cdot R$$

(6) $$\frac{\mathrm{dD}}{\mathrm{dt}}=b_{D}+\frac{a_{D}}{1+{\left(K_{D}\cdot R\right)}^{2}}-\text{μ}\cdot D$$

(7) $$\frac{\mathrm{dA}}{\mathrm{dt}}=\frac{k_{\mathrm{cat},D}\cdot \text{OA}}{K_{m,D}+\text{OA}}\cdot D-\frac{k_{\mathrm{cat},B}\cdot A}{K_{m,B}+A}\cdot B-2(k_{f}\cdot R\cdot A^{2}-k_{r}\cdot \text{aR})-\text{μ}\cdot A$$

(8) $$\frac{d\text{aR}}{\mathrm{dt}}=k_{f}\cdot R\cdot A^{2}-k_{r}\cdot \text{aR}-\text{μ}\cdot \text{aR}$$

where *R*, *D*, *A*, and aR represent the concentrations of transcription factor FadR, uptake enzyme FadD, internalized fatty acid acyl-CoA, and sequestered complex acyl-CoA-FadR, respectively (Fig. 1B). The reversible sequestering of one FadR dimer by two acyl-CoA molecules (stoichiometry as defined in reference 35) is modeled as mass-action kinetics in the term *k_f_RA*^2^ − *k_r_aR*. The term *P_R_*(*R*, *p_r_*) represents the expression and autoregulation of the *fadR* promoter. To model FadR negative autoregulation for the wild-type strain, we use equation 9:

(9) $$P_{R,n}=b_{n}+\frac{a_{n}}{1+K_{n}\cdot R}$$

To fit model parameters, we first extended the model to simulate batch culture and then applied a least-squares fitting of simulations to time course measurements of RFP fluorescence expressed under an fadD promoter, from the Δ*fadE* mutant strain, in various concentrations of oleic acid (see details in Text S1 and Table S2A). The fitting results are illustrated in Fig. S1, and the fitted parameter values are reported in Table S2B. These values were used throughout this study, unless otherwise stated. To understand the impact of the model parameters on the recovery time, we performed global parameter sensitivity analysis (details in Text S1 and Fig. S4). To model the strains with positive autoregulation and constitutive expression of FadR, we use equations 10 and 11:

(10) $$P_{R,p}=b_{p}+\frac{a_{p}\cdot K_{p}\cdot R}{1+K_{p}\cdot R}$$

(11) $$P_{R,c}=p_{c}$$

10.1128/mBio.03112-19.2 FIG S1 (A) Plot of time-series data of measured optical density (OD), in log scale, during growth in medium induced with titrations of oleic acid. Values of the fitted growth rate to each data series are given in the inset, including average growth rate (based on all data series) used in the model; modeled growth is shown with a red line ± the SEM with red dashed lines. (B) Ensemble of 100 independent fits (gray curves) and the optimal fit (colored curves) of simulations to time course data (point with error bars) after converting fluorescence values to concentration. Error bars in the data represent the SEM from biological triplicates (*n* = 3). Fitting performance is in the inset. Download FIG S1, PDF file, 0.7 MB. Copyright © 2020 Hartline et al. 2020 Hartline et al. This content is distributed under the terms of the Creative Commons Attribution 4.0 International license.

10.1128/mBio.03112-19.3 FIG S2 (A) Exposure time affects recovery dynamics. (B) Acyl-CoA accumulates to steady state, but rise time generally falls for increases in consuming enzyme (inset). (C) Amount of FadR released from sequestered complex after 1 doubling time in the off state, after various lengths of exposure to oleic acid. (D) Amount of FadR in sequestered complex accumulates for longer exposure times. (E) Recovery times are affected by the amount of FadR sequestered in complex. All dynamics are shown for four 6, 15.3, 39 and 100 μM consuming enzyme. Download FIG S2, PDF file, 0.1 MB. Copyright © 2020 Hartline et al. 2020 Hartline et al. This content is distributed under the terms of the Creative Commons Attribution 4.0 International license.

10.1128/mBio.03112-19.4 FIG S3 Characterization and use of PA reporter strain. (A) Dose response of P_fadRpo_-rfp indicates that FadR activated and OA inhibited P*_fadRpo_* expression. Error bars are the SEM for biological replicates (*n* = 3), and the blue curve is the fit of a Hill equation to the mean oleic acid concentrations. (B) Time course fluorescence data for the switching experiment of the PA reporter strain. Cells were induced by 1 mM oleic acid at time zero and grown for three different exposure times, 3, 6, and 9 h (dashed vertical lines), after which cultures were rapidly switched to fresh medium lacking oleic acid (off state). Error bars represent the SEM of the results from biological triplicates. Download FIG S3, PDF file, 0.1 MB. Copyright © 2020 Hartline et al. 2020 Hartline et al. This content is distributed under the terms of the Creative Commons Attribution 4.0 International license.

10.1128/mBio.03112-19.5 FIG S4 Global sensitivity analysis of recovery time to model parameters. Shown is a bar plot of the total-order sensitivity indices calculated from global sensitivity analysis (GSA) with eFAST. Sensitivities were calculated from 257 samples per search curve (set of parameters), and this sampling was repeated 7 times to ensure coverage of parameter values. Bars and error bars show the average and standard deviation of sensitivities over the 7 repeated samplings. eFAST assigns a dummy parameter a small, nonzero sensitivity (last bar). This was exploited to perform a two-tailed *t* test to calculate whether the sensitivity of each parameter was significantly greater than that of the dummy parameter (*, using a significance *α* = 0.01). Parameters are listed in descending order of sensitivity (bottom). Download FIG S4, PDF file, 0.1 MB. Copyright © 2020 Hartline et al. 2020 Hartline et al. This content is distributed under the terms of the Creative Commons Attribution 4.0 International license.

### Model simulations.

The model was solved with the MATLAB R2018a ODE solver suite. To simulate the on state, simulations were initialized using steady-state values achieved from simulations of the preculture (oleic acid [OA] concentration, 0 μM), and a constant oleic acid concentration was set to 1,000 μM. Simulations were then run for a defined exposure time. To simulate the off state, the system was initialized from the state achieved at the end of the on state, and the oleic acid concentration was set to 0 μM. Simulations were then run to steady state, and recovery times were calculated as the time from the start of the off state until FadD reached halfway between its initial value and minimum steady-state value. To calculate the cost of FadR synthesis in the on and off states (Fig. 4C), we integrated simulations of the FadR synthesis rate over 48 h in each state.

In Fig. 3, for fair comparison, model parameters are set such that the steady-state concentration of FadR is the same for all three architectures prior to switching to the on state. Likewise, in Fig. 4B and C for fair comparison, *fadR* promoter strengths for both architectures were set to achieve the same concentration of sequestered FadR in the on state (and thus equal recovery times).
