# Supplementary material for: Metabolite Sequestration Enables Rapid Recovery from Fatty Acid Depletion in Escherichia coli
Source: mBio. 2020 Mar 17;11(2):e03112-19. doi: 10.1128/mBio.03112-19 (PMC7078478; doi:10.1128/mBio.03112-19)
Supplement: TABLE S1 [file mBio.03112-19-st001.docx]

**Table S1.** List of metabolite-responsive transcription factors (TF) that control expression of nutrient uptake enzymes in *Escherichia coli*, taken from the EcoCyc database. All these systems follow the schematic in Figure 1A.

| **TF** | **Name** | **TF autoregulation** | **Operon inhibited by TF** | **Sequestering metabolite** |
| --- | --- | --- | --- | --- |
| ArsR | Arsenate inducibility regulator | Negative | arsB, from arsRBC operon | Arsenite / Antimonite ion |
| AlsR | Allose utilization regulator | Negative | alsABC, from alsRBACE operon | D-allose |
| BetI | Betaine Inhibitor | Negative | betT | Choline |
| ChbR | Chitobiose regulator | Negative | chbBCA from chbBCARFG operon | N,N’-diacetylchitobiose 6-phosphate |
| CytR | Cytidine regulator | Negative | nupC and nupG | Cytidine |
| FadR | Fatty acid degradation regulon | Negative | fadD | Acyl-CoA |
| GntR | Gluconate repressor | None, constitutive | gntT, gntU | D-Gluconate |
| LacI | Lactose inhibitor | None, constitutive | lacZYA | Allolactose |
| LldR | Lactate regulator | Negative | lldP, from lldPRD operon | S-lactate |
| LsrR | Quorum sensing system | Negative | lsrACDB operon | AI-2 (autoinducer) |
| NagC | N-acetylglucosamine transcriptional regulator | Negative | chbF, from chbBCARFG operon | Acetyl-D-glucosamine 6-phosphate |
| NanR | N-acetyl-neuraminic acid regulator | None, constitutive | nanT, from nanATEK-yhcH operon | N-acetylneuraminate |
| PaaX | Phenylacetic acid regulator | Negative | paaK, from paa operon | Phenylacetyl-CoA |
| PuuR | Putrescine utilization and transport regulator | Negative | puuP, from puuAP operon | Putrescine |
| RbsR | Ribose repressor | Negative | rbsACB, from rbs operon | D-ribose |
| SrlR | Glucitol Repressor | Negative | srlAEB, from srlAEBD-gutM-slrR-gutQ operon | D-sorbitol |
| TreR | Trehalose repressor | None, constitutive | treB, from treBC operon | Trehalose 6-phosphate |
| UlaR | Utilization of l-ascorbic acid repressor | None, constitutive | ulaABC, from ulaABCDEF operon | L-ascorbate 6-phosphate |
