# Supplementary material for: Metabolite Sequestration Enables Rapid Recovery from Fatty Acid Depletion in Escherichia coli
Source: mBio. 2020 Mar 17;11(2):e03112-19. doi: 10.1128/mBio.03112-19 (PMC7078478; doi:10.1128/mBio.03112-19)
Supplement: TEXT S1 [file mBio.03112-19-s0001.docx]

**Supplementary Methods S1**

**Metabolite sequestration enables rapid recovery from fatty acid depletion in *Escherichia coli***

Christopher J. Hartline^1,†^, Ahmad A. Mannan^2†^, Di Liu^1^, Fuzhong Zhang^1,*^, Diego A. Oyarzún^3,4,*^

^1^ Department of Energy, Environmental & Chemical Engineering, Washington University in St Louis, St. Louis, 63130, USA

^2^ Warwick Integrative Synthetic Biology Centre & School of Engineering, University of Warwick, Coventry CV4 7AL, UK

﻿^3^ School of Informatics, University of Edinburgh, Edinburgh EH8 9AB, UK

^4^ School of Biological Sciences, University of Edinburgh, Edinburgh EH9 3BF, UK

^†^ Equal contribution

^*^ Corresponding authors: F. Zhang ([fzhang@seas.wustl.edu](mailto:fzhang@seas.wustl.edu)) and D. A. Oyarzún ([d.oyarzun@ed.ac.uk](mailto:d.oyarzun@ed.ac.uk)).

**Table of Contents**

**1** Kinetic model of fatty acid uptake

**2** Impact of exposure time to nutrient

**3** Steady state analysis: autoregulation affects FadR levels during induction

**4** Construction and characterization of strain with positively autoregulated *fadR*

**5** Steady state analysis: impact of promoter strength on sequestered FadR

**6** Sensitivity analysis of kinetic model

**1. Kinetic model of fatty acid uptake**

To model the system in Figure 1(B), we use the kinetic model:

|  | [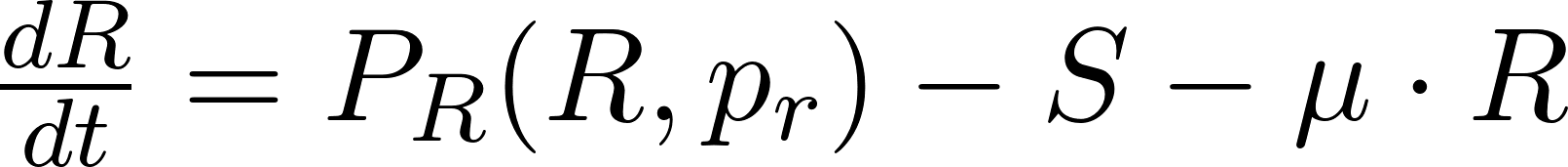](about:blank), | (E1) |
| --- | --- | --- |
|  | [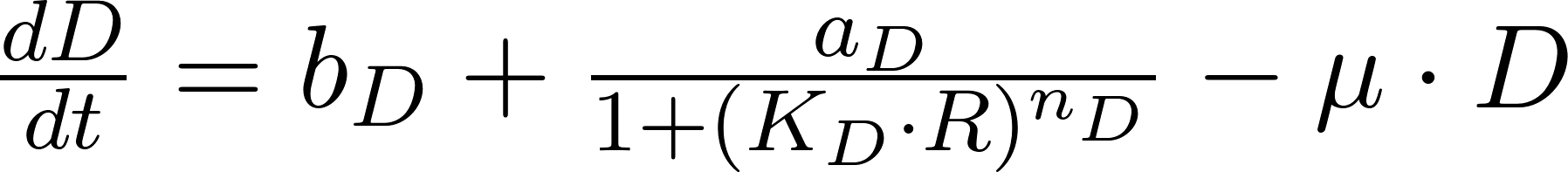](about:blank), | (E2) |
|  | [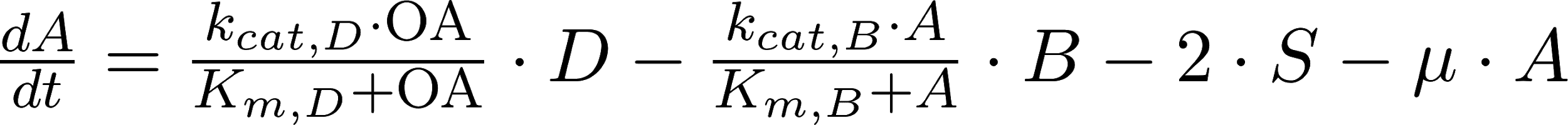](about:blank), | (E3) |
|  | [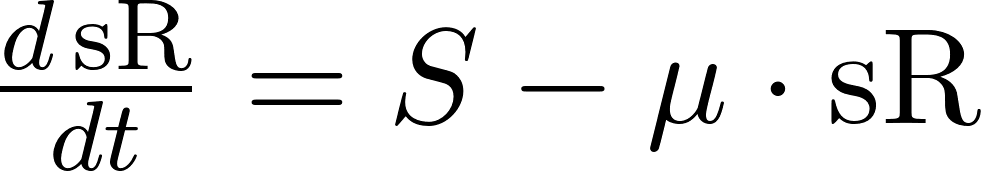](about:blank), | (E4) |
|  | 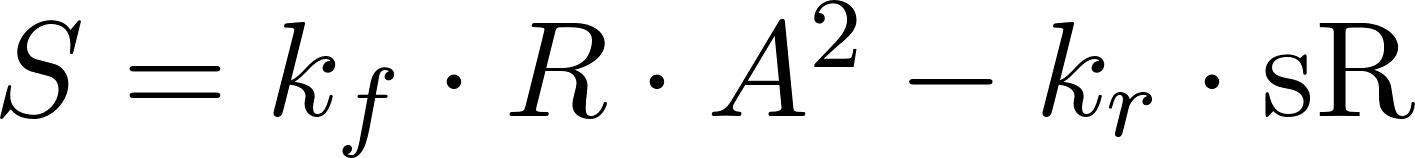, | (E5) |

where *R*, *D*, *A* and aR represent the concentrations of transcription factor FadR, uptake enzyme FadD, internalized fatty acyl-CoA and sequestered acyl-CoA-FadR complex, respectively (Figure 1B). During inducting, two molecules of acyl-CoA bind to sequester 1 dimer of FadR [2]. We model this reversible binding as mass-action kinetics (Eq. E5). The term [
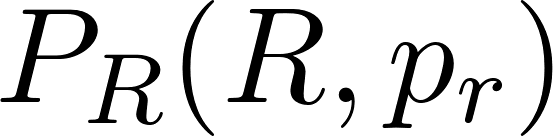
](about:blank) represents the expression and autoregulation of the fadR promoter. To model TF expression when under negative autoregulation (*n*), positive autoregulation (*p*) or constitutive expression (*c*), we write

|  | [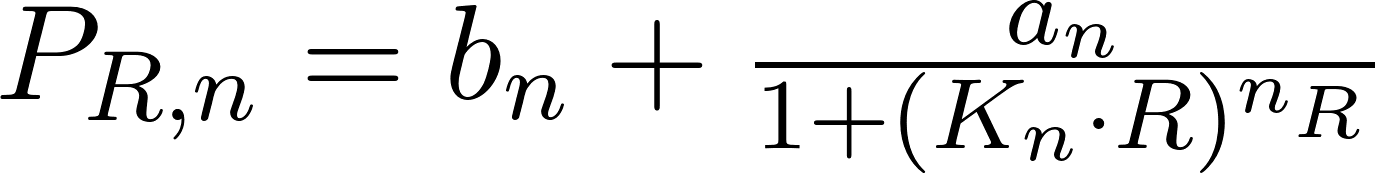](about:blank), | (E6) |
| --- | --- | --- |
|  | [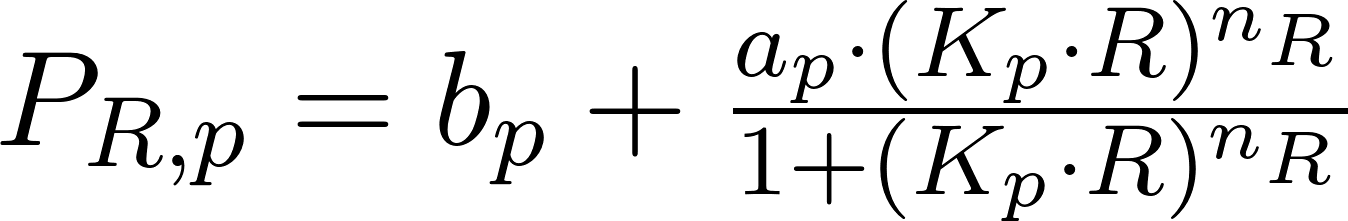](about:blank), | (E7) |
|  | [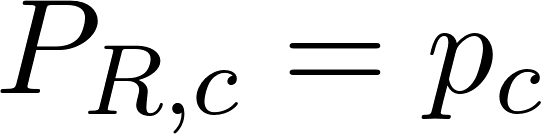](about:blank), | (E8) |

respectively. We can use the model to simulate growth in continuous culture by fixing oleic acid concentration (OA) in Eq. E3. Description of model parameters can be found in Supplementary Table S7A.

To fit model parameters, we use time course data from batch cultures induced with titrations of oleic acid, shown in Figure S2(B). We used a red fluorescent protein (RFP) gene placed at 3’ of the *fadD* promoter on a low copy number plasmid (pSfadDk-RFP). The plasmid was incorporated to a *fadE* knockout strain to make *ΔfadE*-reporter. The *fadE* knockout strain was chosen to reduce the consumption rate of intracellular acyl-CoA and to simplify the metabolite dynamics for this parameterization purpose. Cells were cultivated in M9 glycerol (M9G) medium, in flasks, to exponential growth phase and induced with varying concentrations of oleic acid. Time course measurements of RFP fluorescence (Supplementary Figure S2B) and cell density (Supplementary Figure S2A) were recorded.

For model fitting we extended the Eqs. (E1)-(E8) with population growth in batch culture:

|  | 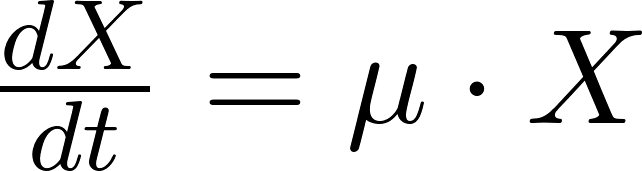, | (E9) |
| --- | --- | --- |
|  | [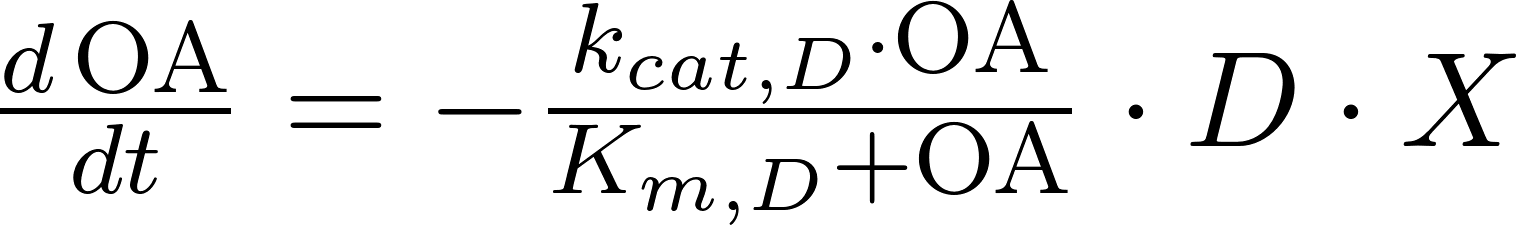](about:blank), | (E10) |

with parameters defined in Supplementary Table S7A. We first converted fluorescence values to units of concentration (μM) by assuming that the average fluorescence value in the absence of inducer (dark blue points, Supplementary Figure S2B) represents the steady state concentration of FadD reported in [3], measured in cells grown in the same media as ours (M9G). This gives a conversion factor of 1.12x10^-4^ μM per unit of fluorescence, which was then applied to all fluorescence values; results are in Supplementary Figure S2B.

We then performed a weighted least-squares fitting of simulations to the data. We define [
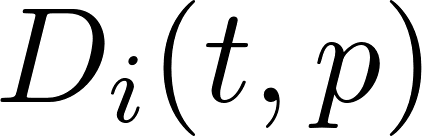
](https://www.codecogs.com/eqnedit.php?latex=D_i(t%2Cp)%250) and [
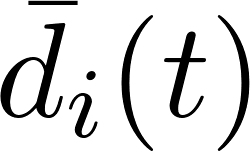
](https://www.codecogs.com/eqnedit.php?latex=%5Cbar%7Bd%7D_i(t)%250) as the simulated and average measured FadD concentration (from three biological replicates), at time t, from the *i*^th^ time series. The index *i* = 1, … ,9 refers to the time course when induced with oleic acid = 0, 0.4, 1, 4, 10, 40, 100, 400, and 1000 μM respectively. Fitting was performed to find optimal values of model parameters (*p*) that minimize the cost function

|  | [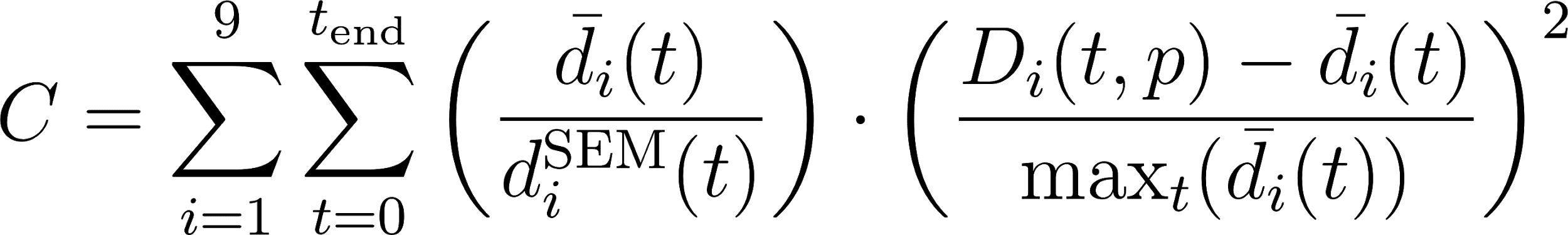](https://www.codecogs.com/eqnedit.php?latex=C%20%3D%20%5Csum%5E%7B9%7D_%7Bi%20%3D%201%7D%20%5Csum%5E%7Bt_%7B%5Ctext%7Bend%7D%7D%7D_%7Bt%20%3D%200%7D%20%5Cleft(%20%5Cfrac%7B%5Cbar%7Bd%7D_i(t)%7D%7Bd%5E%7B%5Ctext%7BSEM%7D%7D_i(t)%7D%20%5Cright)%20%5Ccdot%20%5Cleft(%20%5Cfrac%7BD_i(t%2Cp)%20-%20%5Cbar%7Bd%7D_i(t)%7D%7B%5Cmax_%7Bt%7D%20(%5Cbar%7Bd%7D_i(t))%7D%20%5Cright)%5E%7B2%7D%250), | (E11) |
| --- | --- | --- |

given constraints

|  | [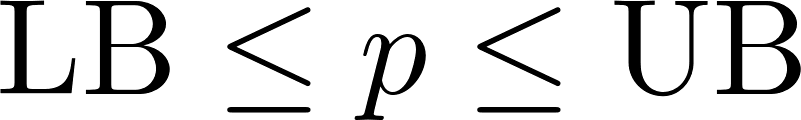](https://www.codecogs.com/eqnedit.php?latex=%5Ctext%7BLB%7D%20%5Cle%20p%20%5Cle%20%5Ctext%7BUB%7D%250), | (E12) |
| --- | --- | --- |

where LB and UB are lower and upper bounds on the parameter search space. The term [
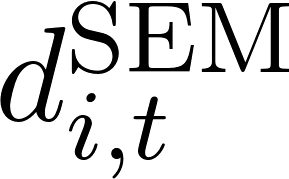
](https://www.codecogs.com/eqnedit.php?latex=d%5E%7B%5Ctext%7BSEM%7D%7D_%7Bi%2Ct%7D%250) is the standard error measured from triplicate data. The term [
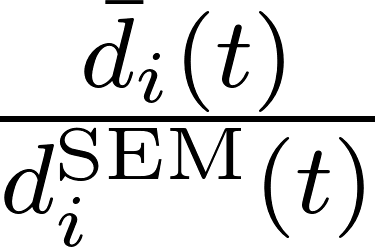
](https://www.codecogs.com/eqnedit.php?latex=%5Cfrac%7B%5Cbar%7Bd%7D_i(t)%7D%7Bd%5E%7B%5Ctext%7BSEM%7D%7D_i(t)%7D%250) in Eq. E11 ensures that the difference between simulation and data at each time point is weighted by the inverse of the relative standard error. This increases the weight of those contributions to the cost where data has a lower measured error. We optimized parameters with a two-step approach. We first used a genetic algorithm (GA) from the Global Optimization toolbox in MATLAB 2018a to find a candidate for a global minimum (using 200 generations of the GA), and then to initialize the solver fmincon and perform a local optimization using the same cost function (Eq. E11). Fitting was performed independently 100 times; results are shown in Figure S2(B), and summary statistics of parameter values are given in Supplementary Table S7B.

Growth rates (*μ*) were estimated through a least-squares fitting of the measured optical densities in Supplementary Figure S2A to the exponential function:

|  | [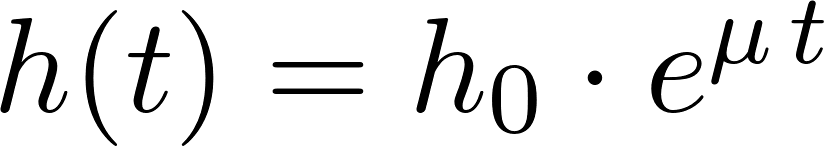](about:blank). | (E13) |
| --- | --- | --- |

To understand the impact of each model parameter on the recovery time, we conducted global parameter sensitivity analysis; see Supplementary S1, part 6, and Figure S5 for details.

**2. Impact of the exposure time to nutrient**

As seen in Supplementary Figure S3, simulations suggest that for small increases in exposure time to oleic acid cause a decrease in recovery time, but for longer times recovery time is increased again. Further analysis of the simulations indicates that for exposure times, acyl-CoA accumulates to higher levels. In the OFF-state larger pools of accumulated acyl-CoA take longer to consume, which causes delays in the release of free FadR in the OFF-state. We infer that this delays the recovery of FadD, increasing recovery time. We hypothesized that the bottleneck lies in the consumption rate of acyl-CoA, as it is limited by the effective *v*_max_ of the consuming enzyme kinetics (in our system *v*_max_ = *k*_cat,B_PlsB). To computationally test this hypothesis, we increased the concentration of consuming enzyme (PlsB) in the model and found a faster release of free FadR which, in turn, reduced recovery time. Since longer exposure times increase the level of FadR stored in complex (a-R), we also found a general decrease in recovery time.

**3. Steady state analysis: autoregulation affects FadR levels during induction**

Here, we ask how the mode of FadR autoregulation affects how the pool of total FadR changes for long exposure times. To quantify this change, we look at the difference in the steady state [
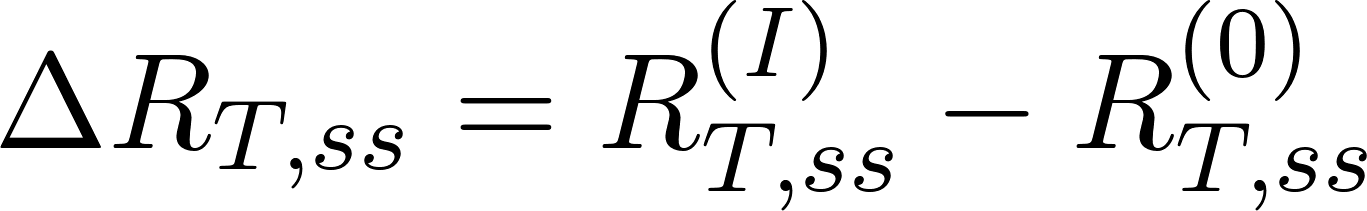
](https://www.codecogs.com/eqnedit.php?latex=%5CDelta%20R_%7BT%2Css%7D%20%3D%20R%5E%7B(I)%7D_%7BT%2Css%7D%20-%20R%5E%7B(0)%7D_%7BT%2Css%7D%250) between the concentration of total FadR achieved in the ON-state ([
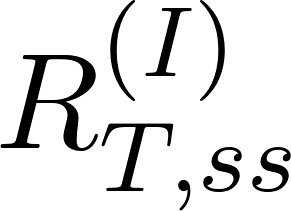
](https://www.codecogs.com/eqnedit.php?latex=R%5E%7B(I)%7D_%7BT%2Css%7D%250)) to that achieved before induction ([
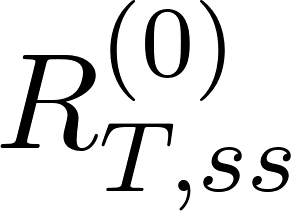
](https://www.codecogs.com/eqnedit.php?latex=R%5E%7B(0)%7D_%7BT%2Css%7D%250)) for each of the three systems (negative autoregulation, positive autoregulation and constitutive expression).

To derive the expression for [
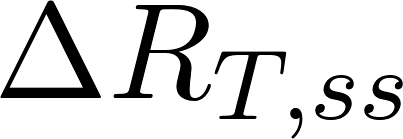
](https://www.codecogs.com/eqnedit.php?latex=%5CDelta%20R_%7BT%2Css%7D%250), we define total FadR as [
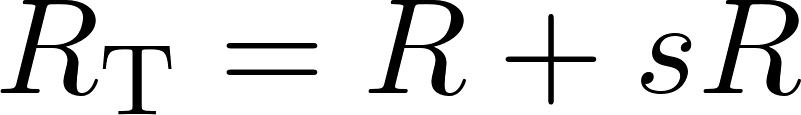
](https://www.codecogs.com/eqnedit.php?latex=R_%7B%5Ctext%7BT%7D%7D%20%3D%20R%20%2B%20sR%250), which from Eq. E1 and E4 follows

|  | [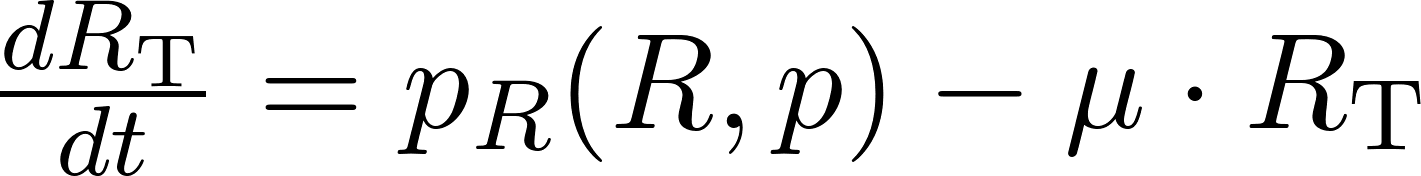](https://www.codecogs.com/eqnedit.php?latex=%5Cfrac%7Bd%20R_%7B%5Ctext%7BT%7D%7D%7D%7Bdt%7D%20%3D%20p_R(R%2Cp)%20-%20%5Cmu%5Ccdot%20R_%7B%5Ctext%7BT%7D%7D%250), | (E14) |
| --- | --- | --- |

where [
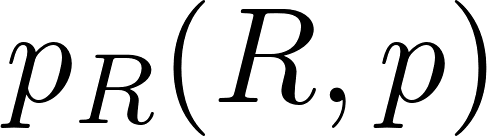
](https://www.codecogs.com/eqnedit.php?latex=p_R(R%2Cp)%250) is the FadR synthesis rate as a function of free FadR and parameters, [
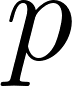
](https://www.codecogs.com/eqnedit.php?latex=p%250), defined in Eqs. E6-E8 for each architecture. [
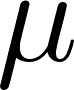
](https://www.codecogs.com/eqnedit.php?latex=%5Cmu%250) is growth rate and assumed constant before and during the ON-state. At steady state Eq. E14 gives

|  | [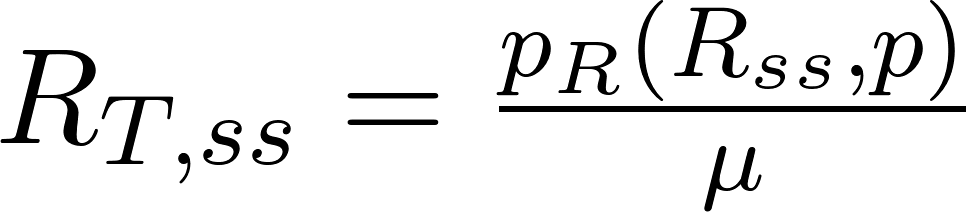](https://www.codecogs.com/eqnedit.php?latex=R_%7BT%2Css%7D%20%3D%20%5Cfrac%7Bp_R(R_%7Bss%7D%2Cp)%7D%7B%5Cmu%7D%250). | (E15) |
| --- | --- | --- |

We can now write down an expression for [
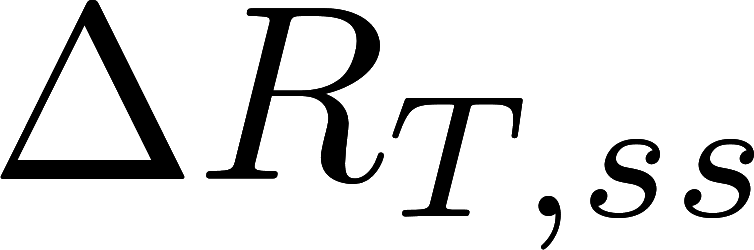
](http://www.texrendr.com/?eqn=%5CDelta%20R_%7BT%2Css%7D%250) as

|  | [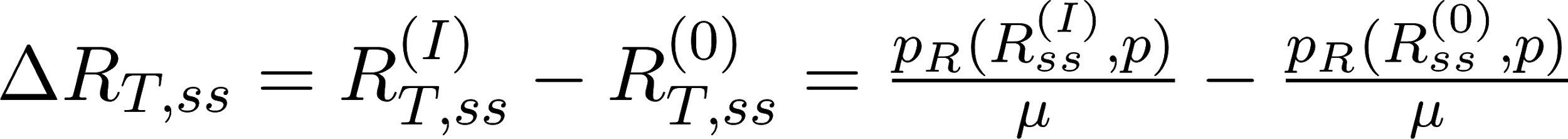](https://www.codecogs.com/eqnedit.php?latex=%5CDelta%20R_%7BT%2Css%7D%20%3D%20R%5E%7B(I)%7D_%7BT%2Css%7D%20-%20R%5E%7B(0)%7D_%7BT%2Css%7D%20%3D%20%5Cfrac%7Bp_R(R%5E%7B(I)%7D_%7Bss%7D%2Cp)%7D%7B%5Cmu%7D%20-%20%5Cfrac%7Bp_R(R%5E%7B(0)%7D_%7Bss%7D%2Cp)%7D%7B%5Cmu%7D%250), | (E16) |
| --- | --- | --- |

where [
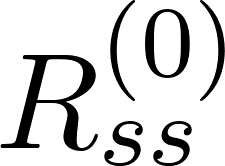
](https://www.codecogs.com/eqnedit.php?latex=R%5E%7B(0)%7D_%7Bss%7D%250) and [
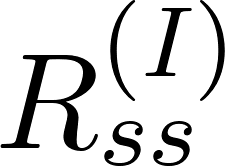
](https://www.codecogs.com/eqnedit.php?latex=R%5E%7B(I)%7D_%7Bss%7D%250) are free FadR before induction and during the ON-state, respectively. In general, Eq. E1 at steady state gives

|  | [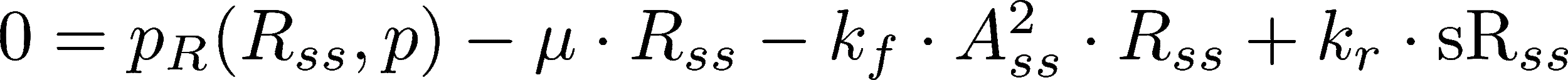](http://www.texrendr.com/?eqn=0%20%3D%20p_R(R_%7Bss%7D%2Cp)%20-%20%5Cmu%20%5Ccdot%20R_%7Bss%7D%20-%20k_f%5Ccdot%20A_%7Bss%7D%5E2%5Ccdot%20R_%7Bss%7D%20%2B%20k_r%5Ccdot%20%5Ctext%7BsR%7D_%7Bss%7D%250), | (E17) |
| --- | --- | --- |

where R_ss_, *A_s_*_s_ and *sR_ss_* are steady state concentrations of free FadR, acyl-CoA and sequestered FadR, respectively. Before induction, we have that [
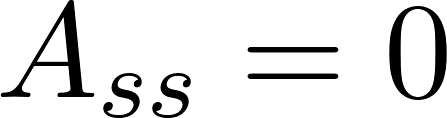
](https://www.codecogs.com/eqnedit.php?latex=A_%7Bss%7D%20%3D%200%250) and [
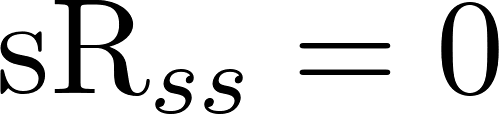
](https://www.codecogs.com/eqnedit.php?latex=%5Ctext%7BsR%7D_%7Bss%7D%20%3D%200%250) for the three architectures. Substitution into Eq. E17 leads to

|  | [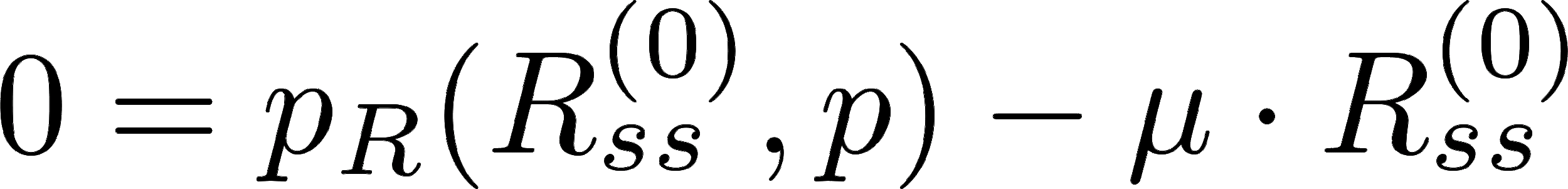](http://www.texrendr.com/?eqn=0%20%3Dp_R(R%5E%7B(0)%7D_%7Bss%7D%2Cp)%20-%20%5Cmu%5Ccdot%20R%5E%7B(0)%7D_%7Bss%7D%250). | (E18) |
| --- | --- | --- |

We now substitute Eqs. E6-E8 into Eq. E18 for each mode of autoregulation, to get:

|  | [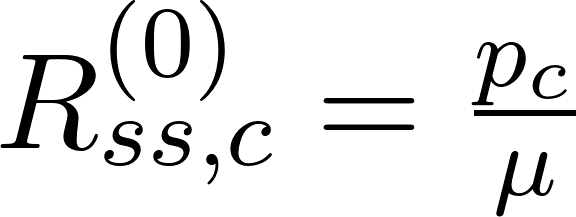](https://www.codecogs.com/eqnedit.php?latex=R%5E%7B(0)%7D_%7Bss%2Cc%7D%20%3D%20%5Cfrac%7Bp_c%7D%7B%5Cmu%7D%250) (constitutive) | (E19) |
| --- | --- | --- |

|  | [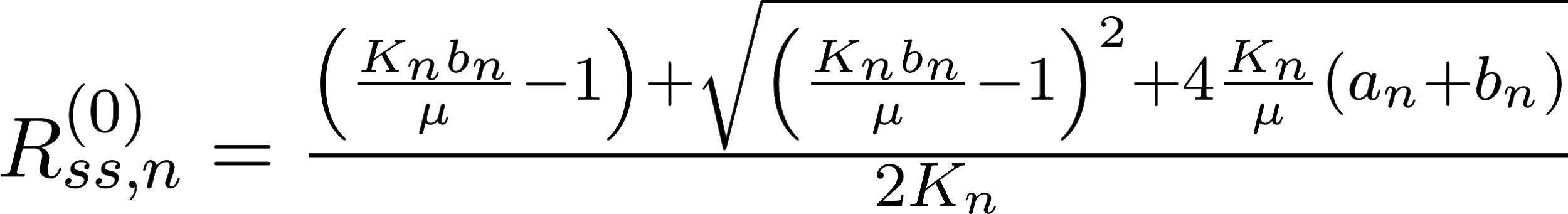](https://www.codecogs.com/eqnedit.php?latex=R%5E%7B(0)%7D_%7Bss%2Cn%7D%20%3D%20%5Cfrac%7B%5Cleft(%20%5Cfrac%7BK_n%20b_n%7D%7B%5Cmu%7D%20-%201%20%5Cright)%20%2B%20%5Csqrt%7B%5Cleft(%20%5Cfrac%7BK_n%20b_n%7D%7B%5Cmu%7D%20-%201%20%5Cright)%20%5E2%20%2B%204%20%5Cfrac%7BK_n%7D%7B%5Cmu%7D%20(a_n%20%2B%20b_n)%7D%7D%7B2%20K_n%7D%250) (negative autoregulation) | (E20) |
| --- | --- | --- |

|  | [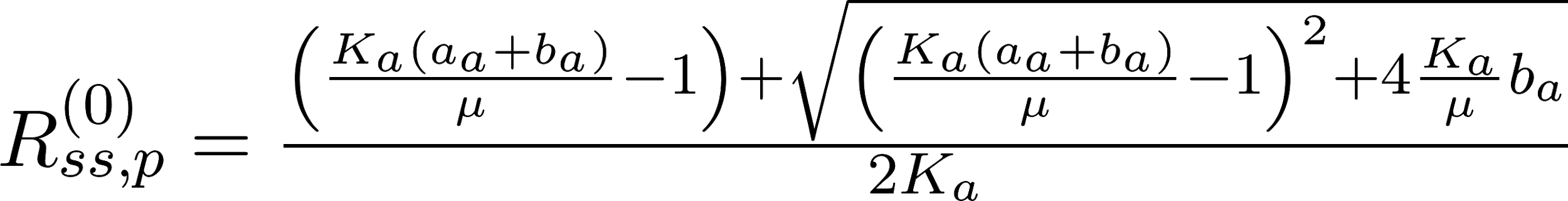](https://www.codecogs.com/eqnedit.php?latex=R%5E%7B(0)%7D_%7Bss%2Cp%7D%20%3D%20%5Cfrac%7B%5Cleft(%20%5Cfrac%7BK_a%20(a_a%20%2B%20b_a)%7D%7B%5Cmu%7D%20-%201%20%5Cright)%20%2B%20%5Csqrt%7B%5Cleft(%20%5Cfrac%7BK_a%20(a_a%20%2B%20b_a)%7D%7B%5Cmu%7D%20-%201%20%5Cright)%20%5E2%20%2B%204%20%5Cfrac%7BK_a%7D%7B%5Cmu%7D%20b_a%7D%7D%7B2%20K_a%7D%250) (positive autoregulation) | (E21) |
| --- | --- | --- |

To compute the steady state during the ON-state, we first solve for *sR* in steady state from Eq. E4 to obtain [
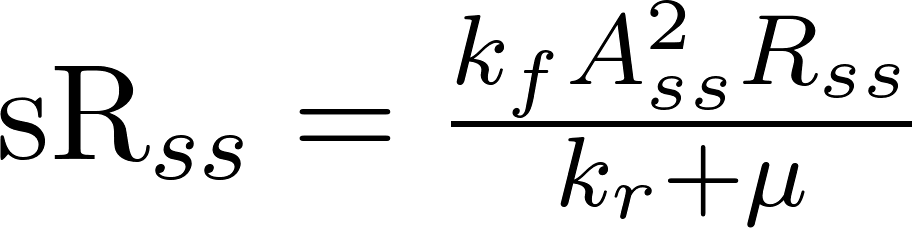
](https://www.codecogs.com/eqnedit.php?latex=%5Ctext%7BsR%7D_%7Bss%7D%20%3D%20%5Cfrac%7Bk_f%20A_%7Bss%7D%5E2%20R_%7Bss%7D%7D%7Bk_r%20%2B%20%5Cmu%7D%250), and then substitute into Eq. E17:

|  | [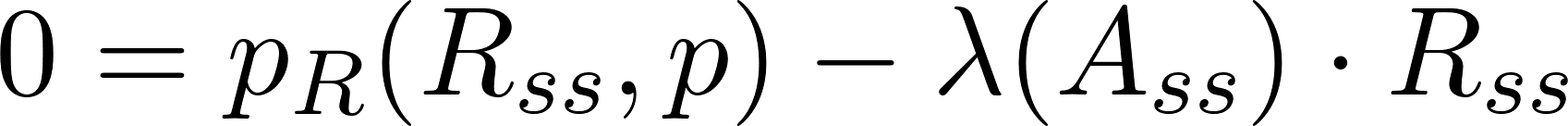](https://www.codecogs.com/eqnedit.php?latex=0%20%3D%20p_R(R_%7Bss%7D%2Cp)%20-%20%5Clambda(A_%7Bss%7D)%20%5Ccdot%20R_%7Bss%7D%250), | (E22) |
| --- | --- | --- |

where we have defined

|  | [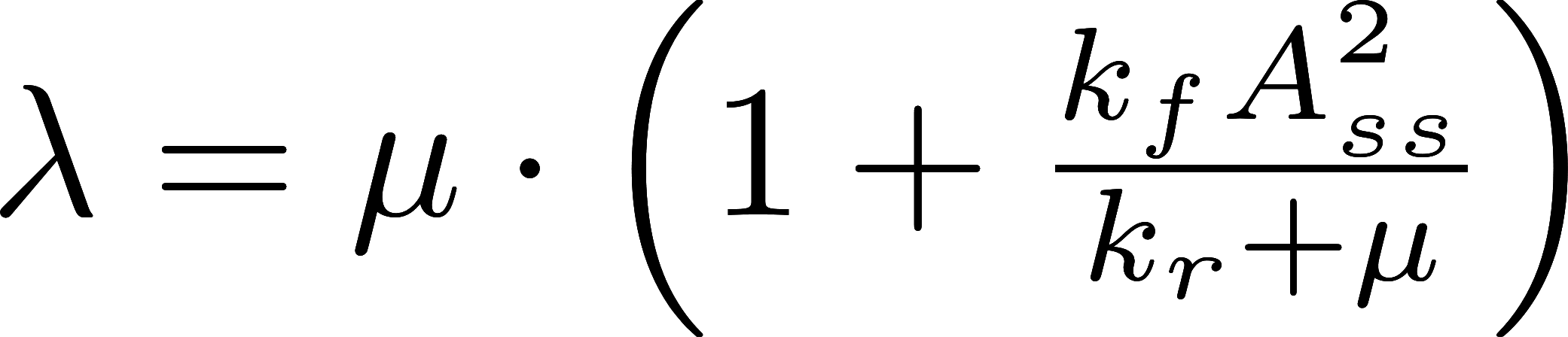](http://www.texrendr.com/?eqn=%5Clambda%20%3D%20%5Cmu%5Ccdot%20%5Cleft(%201%20%2B%20%5Cfrac%7Bk_f%20A_%7Bss%7D%5E2%7D%7Bk_r%20%2B%20%5Cmu%7D%20%5Cright)%250), | (E23) |
| --- | --- | --- |

We now substitute Eqs. E6-E8 into Eq. E2 for each mode of autoregulation, to get the steady state concentration of *R^(I)^_ss_* for each mode of autoregulation:

|  | [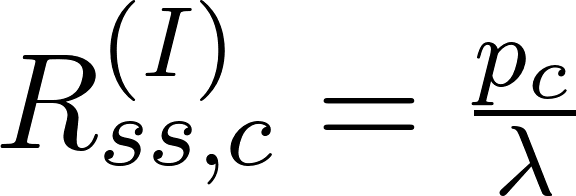](https://www.codecogs.com/eqnedit.php?latex=R%5E%7B(I)%7D_%7Bss%2Cc%7D%20%3D%20%5Cfrac%7Bp_c%7D%7B%5Clambda%7D%250) (constitutive expression) | (E24) |
| --- | --- | --- |
|  | [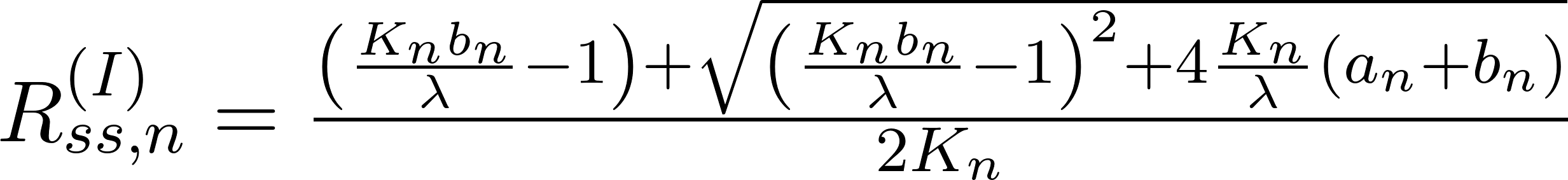](https://www.codecogs.com/eqnedit.php?latex=R%5E%7B(I)%7D_%7Bss%2Cn%7D%20%3D%20%5Cfrac%7B%5Cleft(%20%5Cfrac%7BK_n%20b_n%7D%7B%5Clambda%7D%20-%201%20%5Cright)%20%2B%20%5Csqrt%7B%5Cleft(%20%5Cfrac%7BK_n%20b_n%7D%7B%5Clambda%7D%20-%201%20%5Cright)%20%5E2%20%2B%204%20%5Cfrac%7BK_n%7D%7B%5Clambda%7D%20(a_n%20%2B%20b_n)%7D%7D%7B2%20K_n%7D%250) (negative autoregulation) | (E25) |
|  | [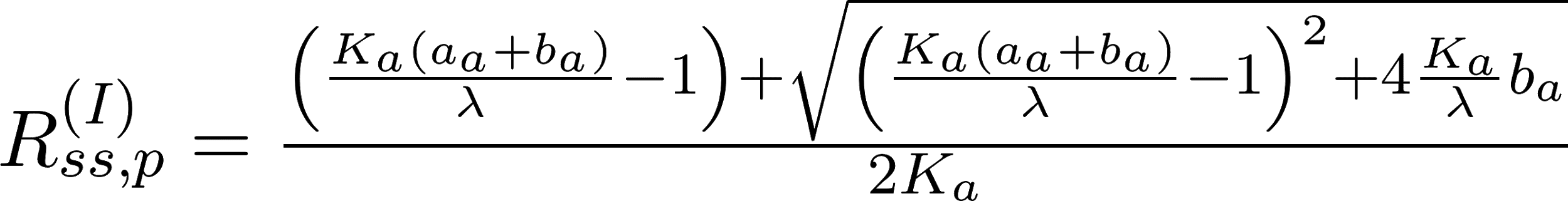](https://www.codecogs.com/eqnedit.php?latex=R%5E%7B(I)%7D_%7Bss%2Cp%7D%20%3D%20%5Cfrac%7B%5Cleft(%20%5Cfrac%7BK_a%20(a_a%20%2B%20b_a)%7D%7B%5Clambda%7D%20-%201%20%5Cright)%20%2B%20%5Csqrt%7B%5Cleft(%20%5Cfrac%7BK_a%20(a_a%20%2B%20b_a)%7D%7B%5Clambda%7D%20-%201%20%5Cright)%20%5E2%20%2B%204%20%5Cfrac%7BK_a%7D%7B%5Clambda%7D%20b_a%7D%7D%7B2%20K_a%7D%250) (positive autoregulation) | (E26) |

We now use the expressions for *R*^(I)^_ss_ in Eqs. E19-E21 and for *R*^(0)^_ss_ in Eqs. E24-E26 to compute the direction of change in steady state concentration of free FadR. From Eq. E23 we have that [
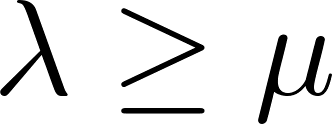
](https://www.codecogs.com/eqnedit.php?latex=%5Clambda%20%5Cge%20%5Cmu%250) for positive parameters, and therefore:

|  | [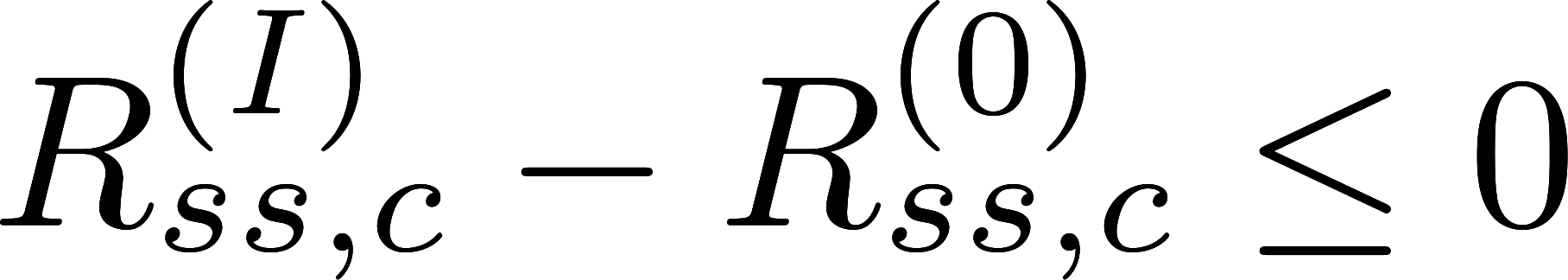](http://www.texrendr.com/?eqn=R%5E%7B(I)%7D_%7Bss%2Cc%7D%20-%20R%5E%7B(0)%7D_%7Bss%2Cc%7D%20%5Cle%200%250), | (E27) |
| --- | --- | --- |
|  | [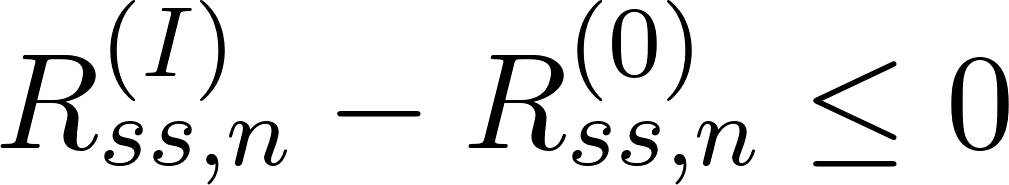](https://www.codecogs.com/eqnedit.php?latex=R%5E%7B(I)%7D_%7Bss%2Cn%7D%20-%20R%5E%7B(0)%7D_%7Bss%2Cn%7D%20%5Cle%200%250), | (E28) |
|  | [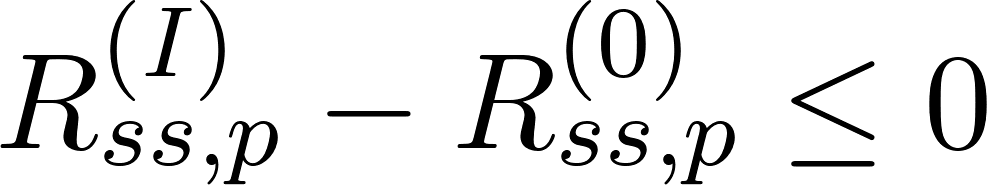](https://www.codecogs.com/eqnedit.php?latex=R%5E%7B(I)%7D_%7Bss%2Cp%7D%20-%20R%5E%7B(0)%7D_%7Bss%2Cp%7D%20%5Cle%200%250). | (E29) |

Therefore, irrespective of the mode of autoregulation, the steady state level of free FadR is always lower in the ON-state relative to its steady state level before induction.

We can now return to Eq. E16 to compute the change in total FadR levels for each mode of autoregulation. **For constitutive expression**, substituting Eq. E8 into E16 we get

|  | [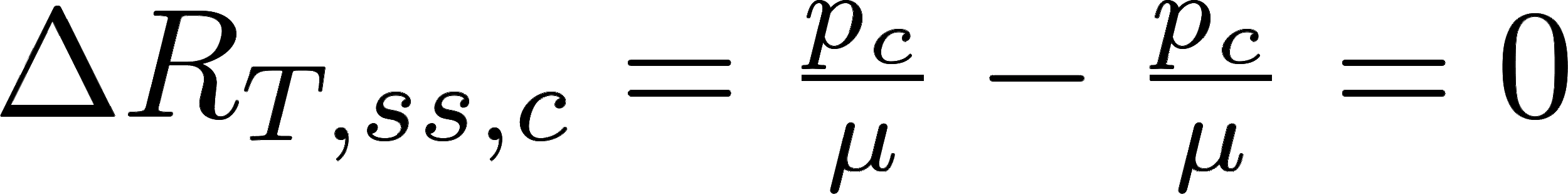](http://www.texrendr.com/?eqn=%5CDelta%20R_%7BT%2Css%2Cc%7D%20%3D%20%5Cfrac%7Bp_c%7D%7B%5Cmu%7D%20-%20%5Cfrac%7Bp_c%7D%7B%5Cmu%7D%20%3D%200%250), | (E30) |
| --- | --- | --- |

and therefore, there is no change in the level of total FadR in the ON-state, relative to its level before induction.

**For negative autoregulation**, substitution of Eq. E6 into E16 gives

|  | [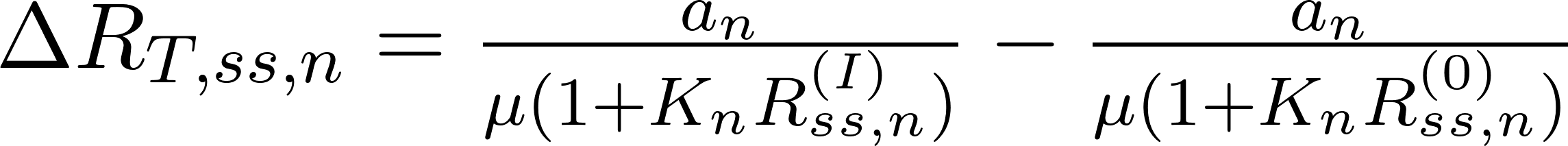](https://www.codecogs.com/eqnedit.php?latex=%5CDelta%20R_%7BT%2Css%2Cn%7D%20%3D%20%5Cfrac%7Ba_n%7D%7B%5Cmu%20(1%20%2B%20K_n%20R%5E%7B(I)%7D_%7Bss%2Cn%7D)%7D%20-%20%5Cfrac%7Ba_n%7D%7B%5Cmu%20(1%20%2B%20K_n%20R%5E%7B(0)%7D_%7Bss%2Cn%7D)%7D%250). | (E31) |
| --- | --- | --- |

Using the relation in Eq. E28, it can be shown that [
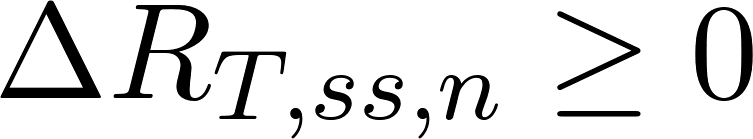
](https://www.codecogs.com/eqnedit.php?latex=%5CDelta%20R_%7BT%2Css%2Cn%7D%20%5Cge%200%250) and therefore the level of total FadR is increased in the ON-state, relative to its level before induction.

**For positive autoregulation**, substitution of Eq. E7 into E16 leads to

|  | [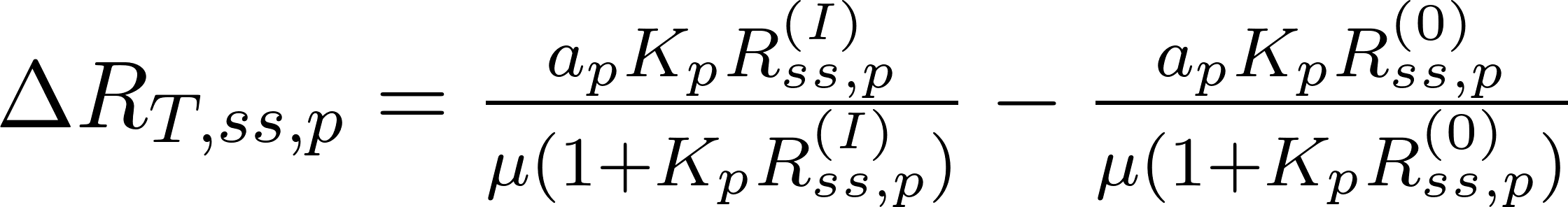](https://www.codecogs.com/eqnedit.php?latex=%5CDelta%20R_%7BT%2Css%2Cp%7D%20%3D%20%5Cfrac%7Ba_p%20K_p%20R%5E%7B(I)%7D_%7Bss%2Cp%7D%7D%7B%5Cmu%20(1%20%2B%20K_p%20R%5E%7B(I)%7D_%7Bss%2Cp%7D)%7D%20-%20%5Cfrac%7Ba_p%20K_p%20R%5E%7B(0)%7D_%7Bss%2Cp%7D%7D%7B%5Cmu%20(1%20%2B%20K_p%20R%5E%7B(0)%7D_%7Bss%2Cp%7D)%7D%250). | (E32) |
| --- | --- | --- |

Using the relation in Eq. E29, it can be shown that [
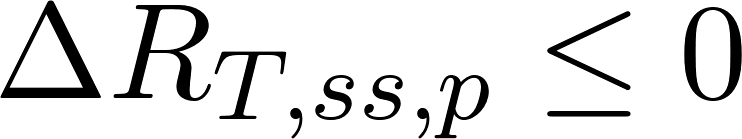
](https://www.codecogs.com/eqnedit.php?latex=%5CDelta%20R_%7BT%2Css%2Cp%7D%20%5Cle%200%250) and therefore level of total FadR is decreased in the ON-state, relative to its level before induction.

In summary, we conclude that:

- For constitutive expression there is no change in total FadR and thus the system maintains the level of sequestered FadR during induction.
- For negative autoregulation, total FadR increases and thus the system builds up a larger pool of sequestered FadR during induction.
- For positive autoregulation, total FadR decreases and thus the system loses sequestered FadR during induction.

**4. Construction and characterization of strain with positively autoregulated *fadR***

To engineer a strain with a self-activating architecture, a portion of the *fadR* promoter sequence, including the -10, -35 and the *fadR* operator sites were replaced with a sequence originating from the promoter of the *fabA* gene, which is positively regulated by FadR. To alter the genome sequence, we utilized pTarget-pCas genome editing system. The original promoter sequence and the engineered promoter (*P_fadR_*_po_) sequence are show in Supplementary Table S9. To enhance the expression of the FadR, we placed a plasmid copy of the positively regulated *fadR*, P*_fadRpo_*-fadR, in a ColE1 origin plasmid. The positively autoregulated reporter strain (named “PA-reporter”) was then created by transforming the pSfadDk-RFP reporter plasmid (Supplementary Table S8).

To confirm the self-activation of *fadR*, we measured the dose-response of the engineered P*_fadRpo_* promoter. A *rfp* gene with a strong ribosome binding site (RBS) was cloned to the 3’ of the P*_fadRpo_* promoter in a BglBrick plasmid (pSfadRpok-rfp). The engineered promoter and RBS sequences are shown in Supplementary Table S9. To measure dose-response output, PA-FadR reporter strain was grown in M9G with oleic acid concentrations = 0, 0.4, 1, 4, 10, 40, 100, 400 and 1000 μM. Cells were grown in a plate reader. Cell culture absorbance and RFP fluorescence were measured. Cultures were initially started at OD_600_ = 0.001 and were allowed to reach steady state. Measurements for each culture condition were made in triplicate. Induction with high concentration of oleic acid reduces the output expression from the P*_fadRpo_* promoter (Supplementary Figure S4B), confirming P*_fadRpo_* as a positively autoregulated promoter.

**5. Steady state analysis: promoter strength affects level of sequestered FadR**

For long exposure times, we have shown that constitutive expression and negative autoregulation can maintain or even build up the concentration of sequestered FadR (see Figure 3 in main text). Here we study how the mode of autoregulation and parameters shape the steady state level of sR (sR_ss_) achieved for long exposure times. We recall from the previous section that the steady state of sequestered FadR in the ON-state is

|  | [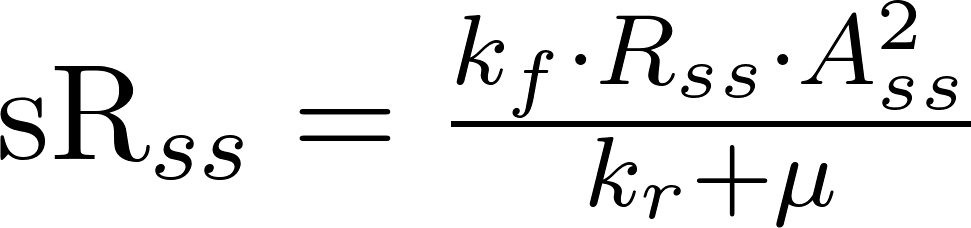](https://www.codecogs.com/eqnedit.php?latex=%5Ctext%7BsR%7D_%7Bss%7D%20%3D%20%5Cfrac%7Bk_f%5Ccdot%20R_%7Bss%7D%5Ccdot%20A_%7Bss%7D%5E2%7D%7Bk_r%20%2B%20%5Cmu%7D%250), | (E33) |
| --- | --- | --- |

where *R*_ss_ is given by the formulae in Eq. E24 and E25 for constitutive and negative autoregulation

|  | [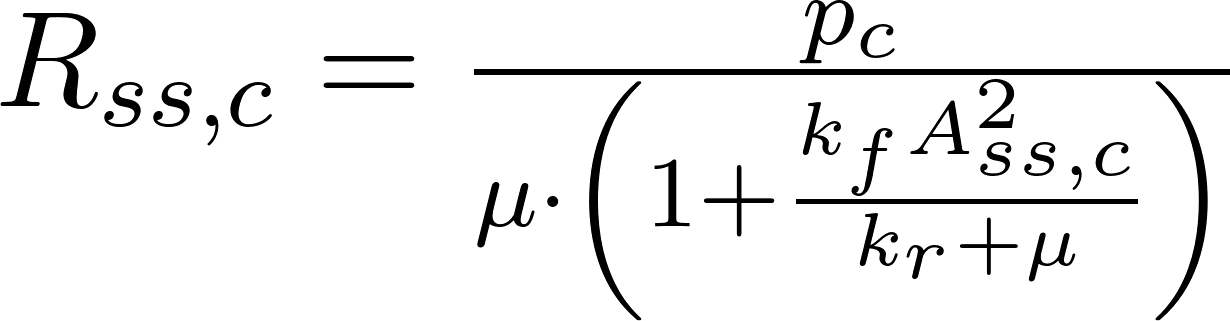](https://www.codecogs.com/eqnedit.php?latex=R_%7Bss%2Cc%7D%20%3D%20%5Cfrac%7Bp_c%7D%7B%5Cmu%5Ccdot%20%5Cleft(%201%20%2B%20%5Cfrac%7Bk_f%20A_%7Bss%2Cc%7D%5E2%7D%7Bk_r%20%2B%20%5Cmu%7D%20%5Cright)%7D%250), | (E34) |
| --- | --- | --- |
|  | [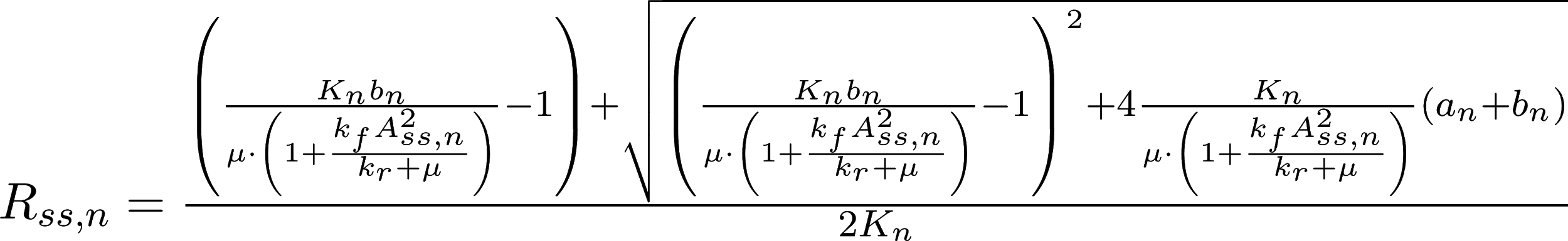](https://www.codecogs.com/eqnedit.php?latex=R_%7Bss%2Cn%7D%20%3D%20%5Cfrac%7B%5Cleft(%20%5Cfrac%7BK_n%20b_n%7D%7B%5Cmu%5Ccdot%20%5Cleft(%201%20%2B%20%5Cfrac%7Bk_f%20A_%7Bss%2Cn%7D%5E2%7D%7Bk_r%20%2B%20%5Cmu%7D%20%5Cright)%7D%20-%201%20%5Cright)%20%2B%20%5Csqrt%7B%5Cleft(%20%5Cfrac%7BK_n%20b_n%7D%7B%5Cmu%5Ccdot%20%5Cleft(%201%20%2B%20%5Cfrac%7Bk_f%20A_%7Bss%2Cn%7D%5E2%7D%7Bk_r%20%2B%20%5Cmu%7D%20%5Cright)%7D%20-%201%20%5Cright)%20%5E2%20%2B%204%20%5Cfrac%7BK_n%7D%7B%5Cmu%5Ccdot%20%5Cleft(%201%20%2B%20%5Cfrac%7Bk_f%20A_%7Bss%2Cn%7D%5E2%7D%7Bk_r%20%2B%20%5Cmu%7D%20%5Cright)%7D%20(a_n%20%2B%20b_n)%7D%7D%7B2%20K_n%7D%250), | (E35) |

respectively. Note that in E34-E35 we have substituted the expression for *λ* given by Eq. E23. Substituting both expressions back into Eq. E33 we get

|  | [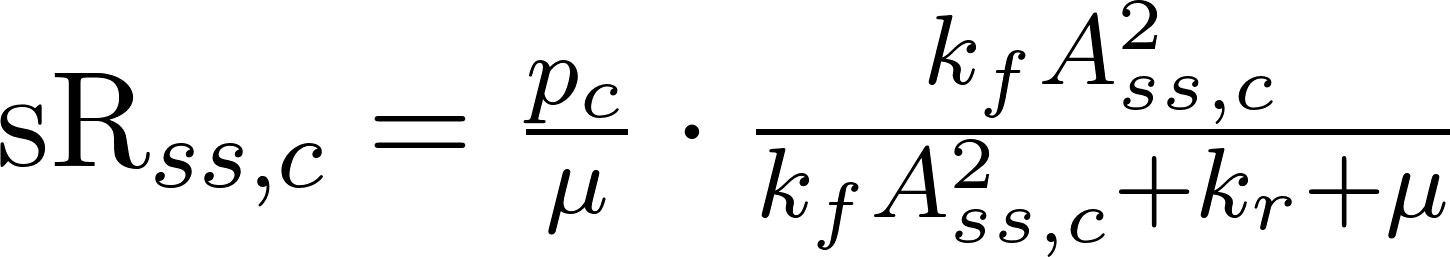](https://www.codecogs.com/eqnedit.php?latex=%5Ctext%7BsR%7D_%7Bss%2Cc%7D%20%3D%20%5Cfrac%7Bp_c%7D%7B%5Cmu%7D%5Ccdot%20%5Cfrac%7Bk_f%20A_%7Bss%2Cc%7D%5E2%7D%7Bk_f%20A%5E2_%7Bss%2Cc%7D%20%2B%20k_r%20%2B%20%5Cmu%7D%250), | (E36) |
| --- | --- | --- |
|  | [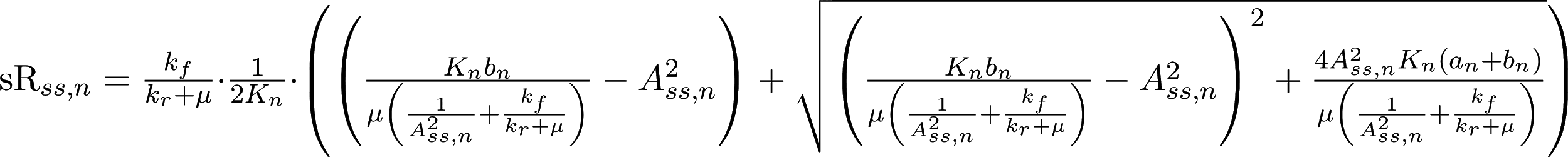](https://www.codecogs.com/eqnedit.php?latex=%5Ctext%7BsR%7D_%7Bss%2Cn%7D%20%3D%20%5Cfrac%7Bk_f%7D%7Bk_r%20%2B%20%5Cmu%7D%5Ccdot%20%5Cfrac%7B1%7D%7B2%20K_n%7D%5Ccdot%20%5Cleft(%20%5Cleft(%20%5Cfrac%7BK_n%20b_n%7D%7B%5Cmu%20%5Cleft(%20%5Cfrac%7B1%7D%7BA%5E2_%7Bss%2Cn%7D%7D%20%2B%20%5Cfrac%7Bk_f%7D%7Bk_r%20%2B%20%5Cmu%7D%20%5Cright)%7D%20-%20A%5E2_%7Bss%2Cn%7D%20%5Cright)%20%2B%20%5Csqrt%7B%5Cleft(%20%5Cfrac%7BK_n%20b_n%7D%7B%5Cmu%20%5Cleft(%20%5Cfrac%7B1%7D%7BA%5E2_%7Bss%2Cn%7D%7D%20%2B%20%5Cfrac%7Bk_f%7D%7Bk_r%20%2B%20%5Cmu%7D%20%5Cright)%7D%20-%20A%5E2_%7Bss%2Cn%7D%20%5Cright)%5E2%20%2B%20%5Cfrac%7B4%20A%5E2_%7Bss%2Cn%7D%20K_n%20(a_n%20%2B%20b_n)%7D%7B%5Cmu%20%5Cleft(%20%5Cfrac%7B1%7D%7BA%5E2_%7Bss%2Cn%7D%7D%20%2B%20%5Cfrac%7Bk_f%7D%7Bk_r%20%2B%20%5Cmu%7D%20%5Cright)%7D%7D%20%20%5Cright)%250), | (E37) |

for constitutive expression and negative autoregulation, respectively. To explore how parameters affect *sR_ss_* for long exposure time, we consider the scenario when there is a large accumulation of acyl-CoA from high levels of inducer in the media. We therefore approximate sR_ss_ for each system by evaluating Eq. E36 and E37 in the limit [
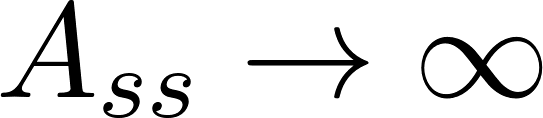
](https://www.codecogs.com/eqnedit.php?latex=A_%7Bss%7D%20%5Crightarrow%20%5Cinfty%250). For constitutive expression we get

|  | [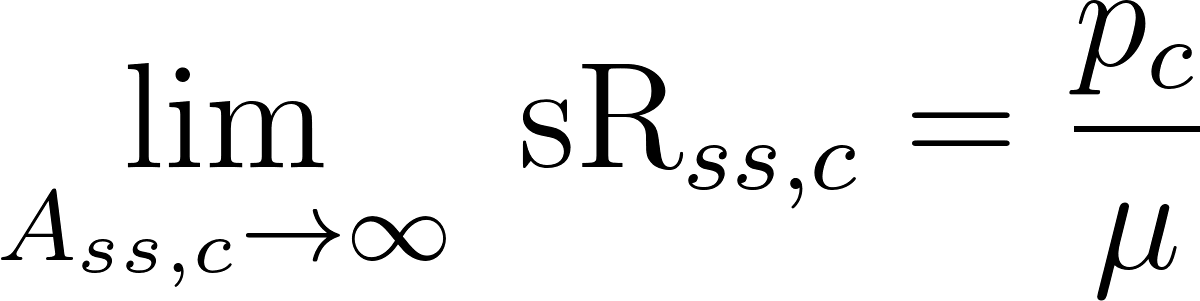](https://www.codecogs.com/eqnedit.php?latex=%5Clim_%7BA_%7Bss%2Cc%7D%20%5Crightarrow%20%5Cinfty%7D%20%5C%3B%20%5Ctext%7BsR%7D_%7Bss%2Cc%7D%20%3D%20%5Cfrac%7Bp_c%7D%7B%5Cmu%7D%250). | (E38) |
| --- | --- | --- |

For negative autoregulation, we further assume that the basal expression of *fadR* promoter is negligible (*b*_n_ = 0), so that Eq. E37 simplifies to

|  | [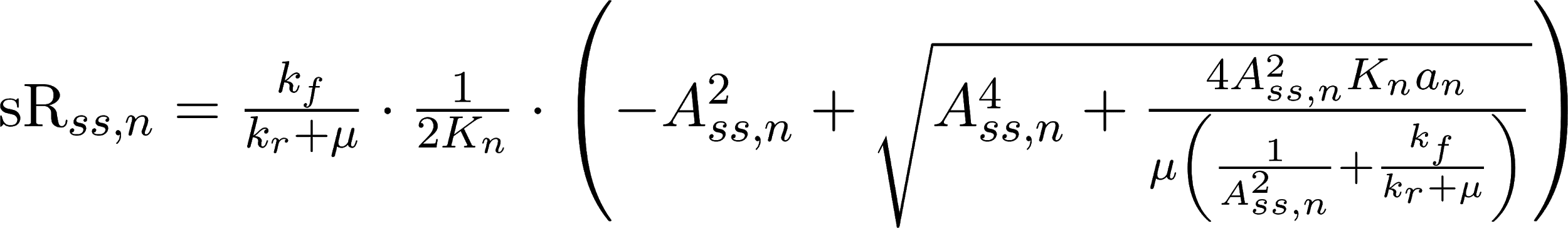](https://www.codecogs.com/eqnedit.php?latex=%5Ctext%7BsR%7D_%7Bss%2Cn%7D%20%3D%20%5Cfrac%7Bk_f%7D%7Bk_r%20%2B%20%5Cmu%7D%5Ccdot%20%5Cfrac%7B1%7D%7B2%20K_n%7D%5Ccdot%20%5Cleft(%20-%20A%5E2_%7Bss%2Cn%7D%20%2B%20%5Csqrt%7B%20A%5E4_%7Bss%2Cn%7D%20%2B%20%5Cfrac%7B4%20A%5E2_%7Bss%2Cn%7D%20K_n%20a_n%7D%7B%5Cmu%20%5Cleft(%20%5Cfrac%7B1%7D%7BA%5E2_%7Bss%2Cn%7D%7D%20%2B%20%5Cfrac%7Bk_f%7D%7Bk_r%20%2B%20%5Cmu%7D%20%5Cright)%7D%7D%20%20%5Cright)%250), | (E39) |
| --- | --- | --- |

and thus we obtain

|  | [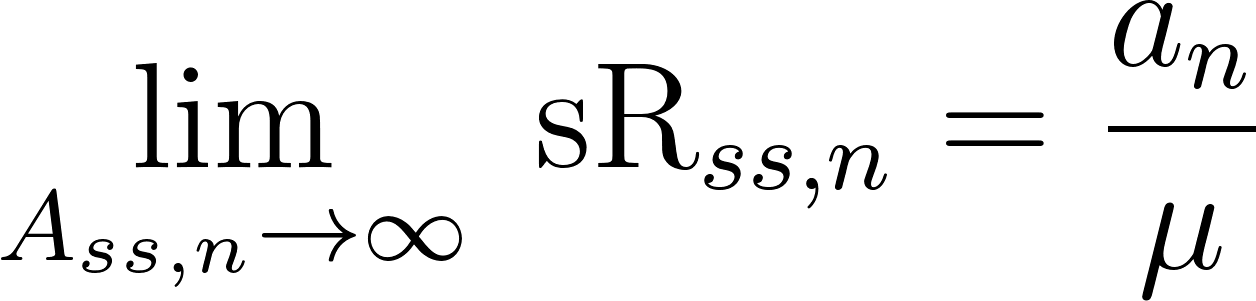](https://www.codecogs.com/eqnedit.php?latex=%5Clim_%7BA_%7Bss%2Cn%7D%5Crightarrow%20%5Cinfty%7D%20%5C%3B%20%5Ctext%7BsR%7D_%7Bss%2Cn%7D%20%3D%20%5Cfrac%7Ba_n%7D%7B%5Cmu%7D%250). | (E40) |
| --- | --- | --- |

In summary, from Eq. E38 and E40 we conclude that in both architectures, sequestered FadR is scaled by the fadR promoter strength.

**6. Sensitivity analysis of kinetic model**

We used global sensitivity analysis (GSA) to quantify the impact of model parameters on the recovery time. We performed GSA using the method of extended Fourier amplitude sensitivity test (eFAST) [4, 5]. We adapted the MATLAB code reported in [5] and implemented eFAST to calculate the first-order and total-order sensitivity indices (Supplementary Figure S5). We assumed that each parameter could vary from 0.1- to 10-fold its fitted value, and model output was defined as the recovery time of FadD for the given input parameters. To enable us to determine which parameters the recovery time was statistically and significantly more sensitive to, we adopted the method of adding a dummy parameter to the model (details in [5]). This was used to compare to the sensitivities of other model parameters and a 2-tailed *t*-test was performed to test for significance. The parameters with significantly higher sensitivities are highlighted with an asterisk in Supplementary Figure S5. The results suggest that recovery time is more sensitive to parameters associated with the sequestering kinetics of FadR by acyl-CoA and the promoter strength of fadR promoter. It is also sensitive to the parameters representing the expression and regulation of FadD, but this is expected as it directly affects recovery time.

**References**

1. Keseler, I.M., et al., *The EcoCyc database: reflecting new knowledge about Escherichia coli K-12.* Nucleic Acids Res., 2017. **45**(D1): p. D543-D550.

2. van Aalten, D.M., et al., *Crystal structure of FadR, a fatty acid-responsive transcription factor with a novel acyl coenzyme A-binding fold.* EMBO J., 2000. **19**(19): p. 5167-5177.

3. Schmidt, A., et al., *The quantitative and condition-dependent Escherichia coli proteome.* Nat. Biotechnol., 2016. **34**(1): p. 104-110.

4. Saltelli, A., S. Tarantola, and K.P. S. Chan, *A Quantitative Model-Independent Method for Global Sensitivity Analysis of Model Output.* Technometrics, 1999. **41**(1): p. 39.

5. Marino, S., et al., *A methodology for performing global uncertainty and sensitivity analysis in systems biology.* J. Theor. Biol., 2008. **254**(1): p. 178-196.
