# Supplementary material for: Metabolite Sequestration Enables Rapid Recovery from Fatty Acid Depletion in Escherichia coli
Source: mBio. 2020 Mar 17;11(2):e03112-19. doi: 10.1128/mBio.03112-19 (PMC7078478; doi:10.1128/mBio.03112-19)
Supplement: TABLE S2 [file mBio.03112-19-st002.docx]

**Table S2. (A)** Parameters of the kinetic model. **(B)** Results of parameter fitting. Optimal parameter values together with search bounds and summary statistics for 100 independent fits. The bounds on growth rate, *μ*, are based on ± two times the SEM from data (shown in Supplementary Fig. S1A, inset). Hill coefficients are fixed to *n_R_*=1 and *n_D_*=2, based on the number of FadR binding sites on the fadR and fadD promoters. Concentration of PlsB is fixed to 0.1369 μM, as taken from Schmidt et al. (2016), Nat. Biotechnol, 34(1).

**(A)**

| **Parameter** | [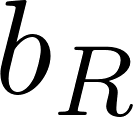](about:blank) | [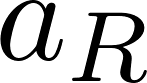](about:blank) | [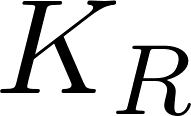](about:blank) | [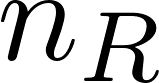](about:blank) |
| --- | --- | --- | --- | --- |
| **Description** | fadR basal exp. rate  Units = [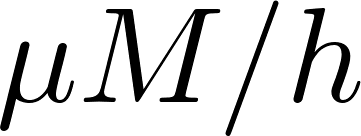](about:blank)  *(Fitted)* | fadR promoter strength  Units = [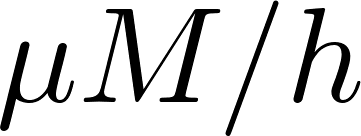](about:blank)  *(Fitted)* | Affinity of FadR for its own promoter  Units = [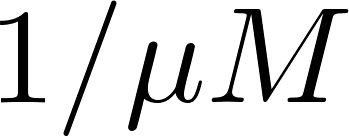](about:blank)  *(Fitted)* | Hill coefficient  Units = N/A  *(Fixed = 1)* |
| **Parameter** | [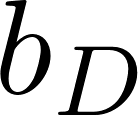](about:blank) | [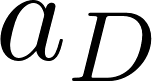](about:blank) | [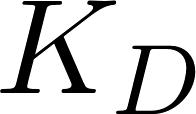](about:blank) | [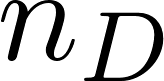](about:blank) |
| **Description** | fadD basal exp. rate  Units = [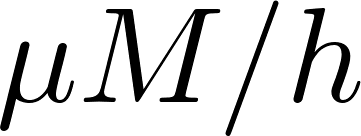](about:blank)  *(Fitted)* | fadD promoter strength  Units = [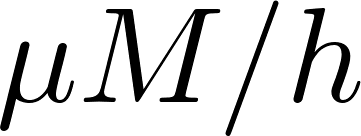](about:blank)  *(Fitted)* | Affinity of FadR for fadD promoter  Units = [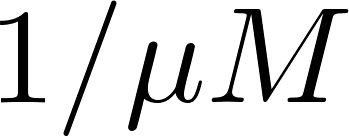](about:blank)  *(Fitted)* | Hill coefficient  Units = N/A  *(Fixed = 2)* |
| **Parameter** | [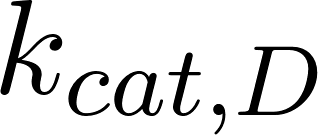](about:blank) | [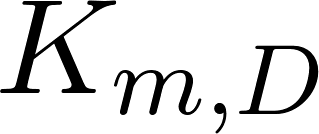](about:blank) | [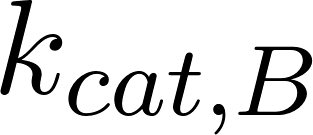](about:blank) | [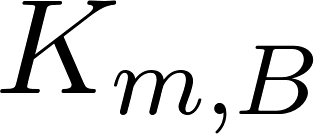](about:blank) |
| **Description** | Turnover rate of FadD  Units = [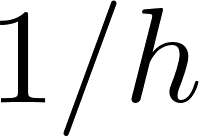](about:blank)  *(Fitted)* | Michaelis const. for FadD  Units = [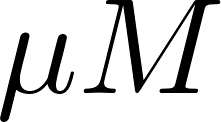](about:blank)  *(Fitted)* | Turnover rate of PlsB enzyme  Units = [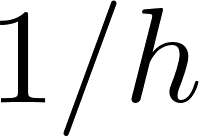](about:blank)  *(Fitted)* | Michaelis const.  Units = [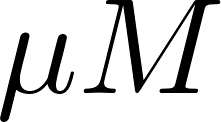](about:blank)  *(Fitted)* |
| **Parameter** | [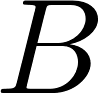](about:blank) | [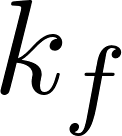](about:blank) | [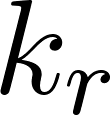](about:blank) | [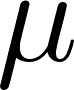](about:blank) |
| **Description** | Conc. of PlsB enzyme  Units = [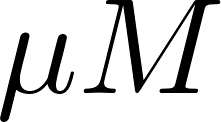](about:blank)  *(Fixed = 0.1369)* | Fwd rate of sequestering  Units = [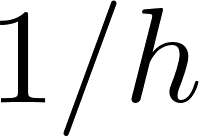](about:blank)  *(Fitted)* | Reverse rate of sequestering  Units = [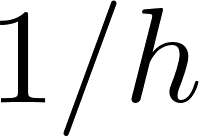](about:blank)  *(Fitted)* | Cell growth rate  Units = [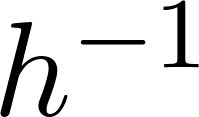](about:blank)  *(Fitted)* |

**(B)**

| **Parameters** | **Optimal** | **Bounds of GA** | | **Summary Statistics** | | | |
| --- | --- | --- | --- | --- | --- | --- | --- |
|  |  | **Lower bound** | **Upper bound** | **Average** | **Median** | **SD** | **CV** |
| *μ* | 0.1818 | 0.1817 | 0.1913 | 0.1854 | 0.1840 | 0.0036 | 1.9172% |
| *b_R_* | 0.0007 | 1.00E-06 | 0.0600 | 0.0210 | 0.0173 | 0.0140 | 66.7954% |
| *a_R_* | 0.0131 | 1.00E-06 | 0.1500 | 0.0414 | 0.0343 | 0.0343 | 82.8857% |
| *K_R_* | 4.3222 | 1.00E-03 | 100.0000 | 27.1436 | 22.9710 | 21.3182 | 78.5385% |
| *n_R_* | 1.0000 | - | - | - | - | - | - |
| *b_D_* | 0.0108 | 1.00E-06 | 0.1000 | 0.0108 | 0.0112 | 0.0015 | 13.5842% |
| *a_D_* | 0.0517 | 1.00E-06 | 0.1000 | 0.0486 | 0.0484 | 0.0021 | 4.4003% |
| *K_D_* | 305.9500 | 1.00E-03 | 750.0000 | 267.2850 | 215.9050 | 201.6395 | 75.4399% |
| *n_D_* | 2.0000 | - | - | - | - | - | - |
| *k_catD_* | 49.0000 | 1.00E-06 | 27,000.0 | 12,364.4 | 12,817.5 | 7,610.1 | 61.5483% |
| *Km_D_* | 0.0672 | 1.00E-02 | 650.0000 | 173.6743 | 149.1950 | 128.8831 | 74.2096% |
| *k_catB_* | 192.9100 | 1.00E-06 | 620.0000 | 235.0742 | 214.5650 | 160.4516 | 68.2557% |
| *Km_B_* | 45,429.0 | 1.00E-02 | 50,000.0 | 29,547.0 | 31,923.5 | 12,371.9 | 41.8719% |
| *PlsB* | 0.1369 | - | - | - | - | - | - |
| *k_f_* | 612.5500 | 1.00E-06 | 625.0000 | 409.3018 | 446.6350 | 174.9435 | 42.7419% |
| *k_r_* | 900.7300 | 1.00E-06 | 3,200.0 | 844.9572 | 515.6600 | 928.0828 | 109.8378% |
| Init. Biomass | 0.1648 | 1.00E-02 | 2.0000 | 0.2308 | 0.1521 | 0.2567 | 111.2037% |
| Obj Value | 82.0500 | - | - | 160,209.7 | 150,475.6 | 161,591.8 | 100.8627% |
