# Supplementary material for: Metabolite Sequestration Enables Rapid Recovery from Fatty Acid Depletion in Escherichia coli
Source: mBio. 2020 Mar 17;11(2):e03112-19. doi: 10.1128/mBio.03112-19 (PMC7078478; doi:10.1128/mBio.03112-19)
Supplement: TABLE S3 [file mBio.03112-19-st003.docx]

**Table S3. (A)** Plasmids used in this study. **(B)** Strains used in this study.

**(A)**

| **Plasmids** | **Replication Origin** | **Operon** | **Resistance** | **Reference** |
| --- | --- | --- | --- | --- |
| pSfadDk-RFP | SC101** | P*_fadD_*-*rfp* | Kan^R^ | This Study |
| pEfadRpoa-fadR | colE1 | P*_fadRpo_-fadR* | Amp^R^ | This Study |
| pSfadRpok-rfp | SC101** | P*_fadR_*_po_-*rfp* | Kan^R^ | This Study |

**(B)**

| **Strains** | **Relevant Genotype** | **Reference** |
| --- | --- | --- |
| *E. coli* DH1 | F- λ- *supE44 hsdR17 recA1 endA1 gyrA96 thi-1 relA1* | Hanahan (1983) J Mol Biol. 166(4) |
| DH1(ΔfadE) | DH1, Δ*fadE* | Steen et al. (2010) Nature 463 |
| WT-reporter | DH1, pSfadDk-RFP | This Study |
| ΔfadE-reporter | DH1, Δ*fadE*, pSfadDk-RFP | This Study |
| PA-reporter | *DH1, fadR::P_fadRpo_-fadR,* pSfadDk-RFP, pEfadRpoa-fadR | This Study |
| PA-FadR reporter | DH1, *fadR::P_fadR_*_po_*-fadR,* pSfadRpok-RFP, pEfadRpoa-fadR | This Study |
