# Supplementary material for: Metabolite Sequestration Enables Rapid Recovery from Fatty Acid Depletion in Escherichia coli
Source: mBio. 2020 Mar 17;11(2):e03112-19. doi: 10.1128/mBio.03112-19 (PMC7078478; doi:10.1128/mBio.03112-19)
Supplement: TABLE S4 [file mBio.03112-19-st004.docx]

**Table S4. Sequences of engineered promoter with positively autoregulated *fadR*.** (A) Native *fadR* promoter sequence, P*_fadR_*. Bold lettering indicates FadR operator site, blue lettering indicates coding sequence. (B) Positively autoregulated *fadR* promoter, P*_fadR_*_po_**_,_** engineered in this work. The underlined sequence is derived from the *fabA* promoter region of *E. coli* DH1 genome. (C) Engineered P*_fadR_*_po_ used to control *rfp* expression.

| (A) | CCCTTTTTCTTCTTTTTGTCTGCTATCAGCGTAGTTAGCC**CTCTGGTATGATGAGTCC**AACTTTGTTTT  GCTGTGTTATGGAAATCTCACTATGGTCATTAAGGCG |
| --- | --- |
| (B) | CCCTTTTTCTTCTTTTTATTCCG**AACTGATCGGACTTGTT**CAGCGTACACGTGTTAGCT  ATCCTGCGTCAACTTTGTTTTGCTGTGTTATGGAAATCTCACTATGGTCATTAAGGCG |
| (C) | CCCTTTTTCTTCTTTTTATTCCG**AACTGATCGGACTTGTT**CAGCGTACACGTGTTAGCTATCCTGCGTCAACTTTGTTTTGCAGGTTTGTAAATAAAGGAGGGAGAAAGGGTATATGGCGAGTAGCGAA |
