# Supplementary material for: Metabolite Sequestration Enables Rapid Recovery from Fatty Acid Depletion in Escherichia coli
Source: mBio. 2020 Mar 17;11(2):e03112-19. doi: 10.1128/mBio.03112-19 (PMC7078478; doi:10.1128/mBio.03112-19)
Supplement: FIG S1 [file mBio.03112-19-sf001.pdf]

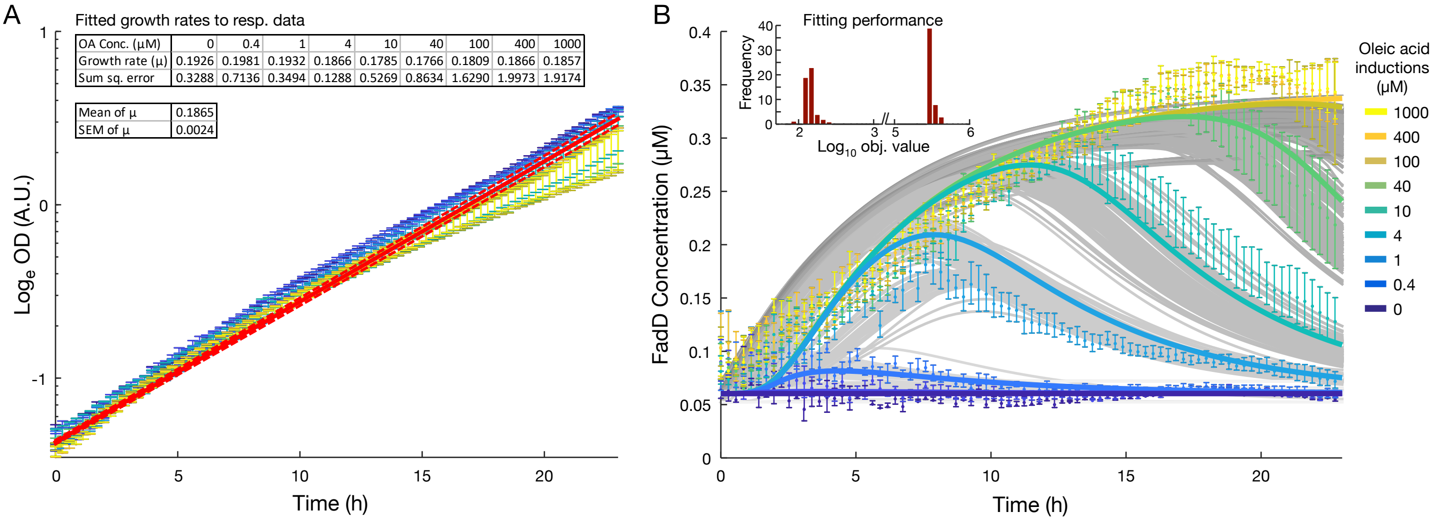

OA Conc. (μM)	0	0.4	1	4	10	40	100	400	1000
Growth rate (μ)	0.1926	0.1981	0.1932	0.1866	0.1785	0.1766	0.1809	0.1866	0.1857
Sum sq. error	0.3288	0.7136	0.3494	0.1288	0.5269	0.8634	1.6290	1.9973	1.9174

Mean of μ	0.1865
SEM of μ	0.0024
