# Supplementary figures and images for: Metabolite Sequestration Enables Rapid Recovery from Fatty Acid Depletion in Escherichia coli
Source: mBio. 2020 Mar 17;11(2):e03112-19. doi: 10.1128/mBio.03112-19 (PMC7078478; doi:10.1128/mBio.03112-19)

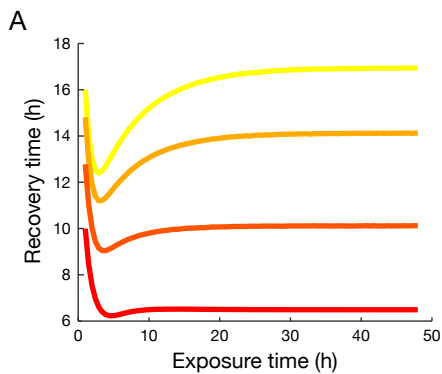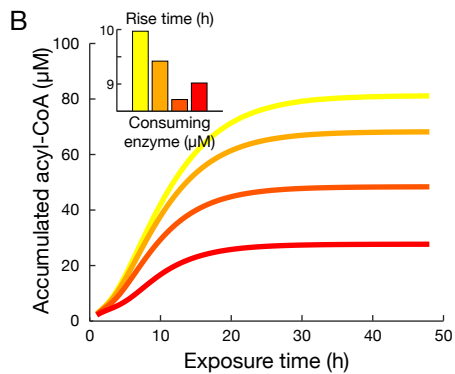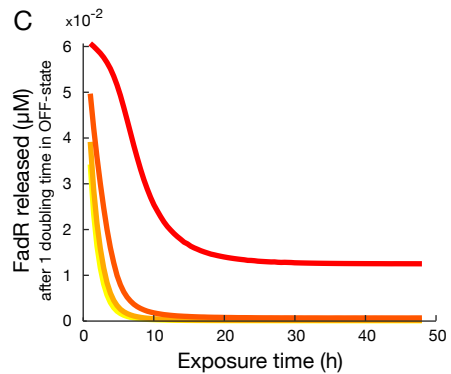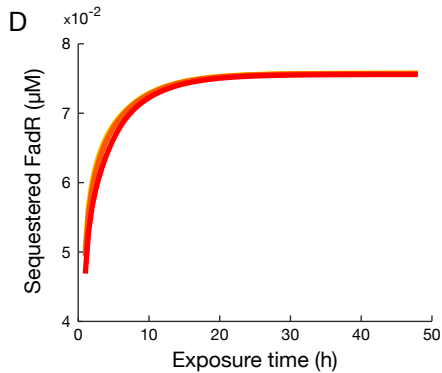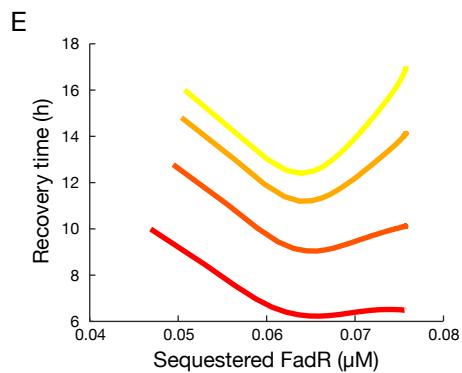

Supplement: FIG S2 [file mBio.03112-19-sf002.pdf]

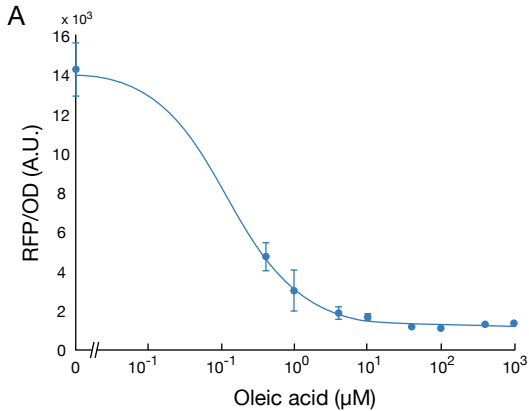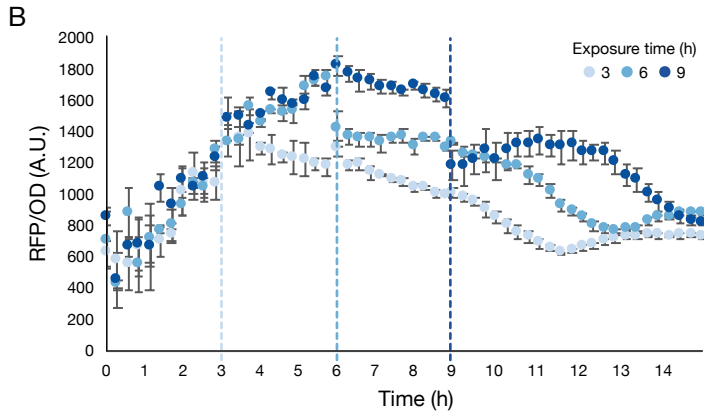

Supplement: FIG S3 [file mBio.03112-19-sf003.pdf]

# Global sensitivity analysis - by eFAST

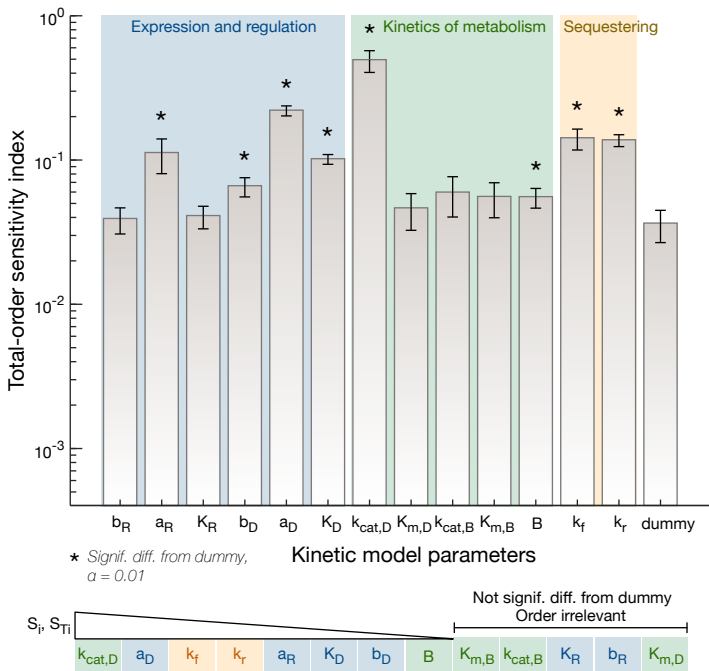

Supplement: FIG S4 [file mBio.03112-19-sf004.pdf]
